# Global Harmonization of Life Cycle Assessments for Small- and Medium-Scale Wastewater Treatment

**DOI:** 10.1021/acsestwater.6c00580

**Published:** 2026-10-01

**Authors:** Emilio Solis, Nils Thonemann, Ranran Wang, Arnold Tukker, Valerio Barbarossa

**Affiliations:** † 4496 Leiden University, Institute of Environmental Sciences (CML), Leiden 2333 CC, the Netherlands; ‡ 12581 Nanjing University, Institute for the Environment and Health, Suzhou 215163, China; § Netherlands Organisation for Applied Scientific Research TNO, Den Haag 2595 DA, the Netherlands; ∥ PBL Netherlands Environmental Assessment Agency, Den Haag 2594 AV, the Netherlands

**Keywords:** wastewater treatment, medium scale, life cycle assessment, harmonization, greenhouse gas emissions, eutrophication

## Abstract

Wastewater management (WWM) is vital for protecting human health and the environment, yet conventional centralized systems are resource-intensive and unsuited to growing urban pressure. Small and medium WWMs offer a potentially more sustainable alternative, but the lack of standardized life cycle assessment (LCA) approaches limits robust comparison and informed decision-making. In this study, we conduct a global harmonization of life cycle assessments of small- and medium-scale systems across 96 LCAs and 9 treatment technologies. By harmonizing life cycle inventories, we eliminate variability in methods, assumptions, and biases, thereby providing a consistent setting to compare environmental impacts. The harmonization process shows that GHG emissions have been underrepresented in inventories. The results suggest that direct emissions to air are a key contributor to climate change, and decarbonization strategies should focus on minimizing these emissions. Sequencing batch reactors showed the lowest average phosphorus and low nitrogen emissions to water with minimal chemical dosing and low direct GHG emissions to air. Our findings support the development of environmentally sustainable small- and medium-scale water-treatment solutions for municipal settings.

## Graphical abstract

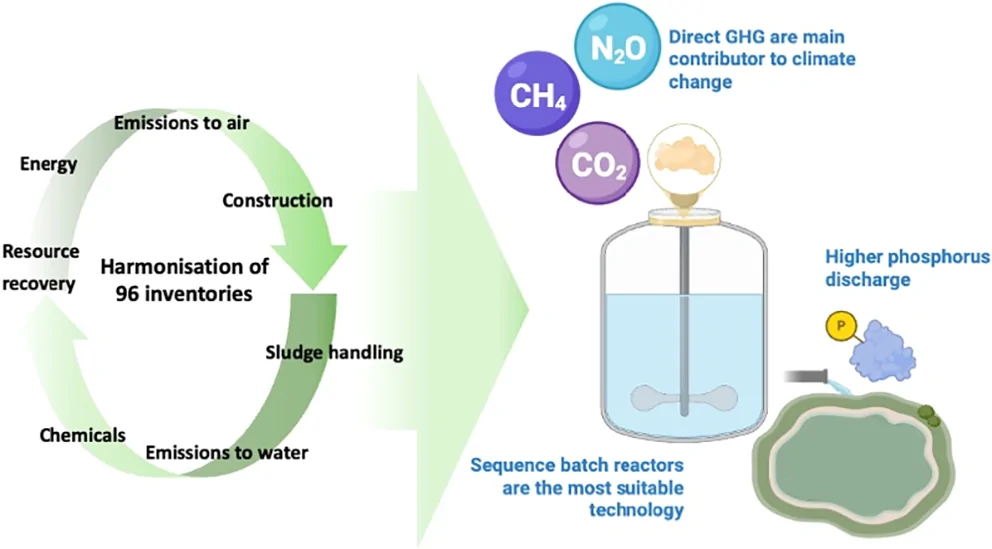

## 1 Introduction

Wastewater treatment (WWM) is crucial to sustain modern societies as it cleans and discharges water back into the environment after it has been used, thereby ensuring good ecological status, sanitation, and public health.^1–3^ However, current approaches are being increasingly scrutinized.^4^ Centralized WWTs (cWWTs) are highly energy-intensive processes and often require large amounts of chemicals, materials, and water.^5^ As centralized systems increase in size to accommodate urban expansion, their maintenance becomes costly and difficult to manage, leading to inefficiencies, leakages, and overflows.^4,6^ There is also an ongoing drive to transform WWM into resource recovery processes, which requires a reimagining of the current treatment paradigm.^7^ Furthermore, in spite of advances in WWM, monitoring data suggest a deterioration in water quality measurements.^8^ Europe is a clear example of this, where there is widespread adoption of WWM yet it displays poor freshwater quality.^9^

Small- and medium-scale WWMs are seen as a potential alternative to improve the environmental, social, and economic performance of large-scale centralized systems and as a modular solution in peri-urban and urban areas to increase the flexibility and functionality of the water system and reduce its associated environmental impacts.^5,7,10^ Traditionally, small- and medium-scale systems were simple and typically used in rural or remote areas, where they achieved modest pollutant removal.^3,11^ Nevertheless, the increase in water discharge standards and technological advances have resulted in a growing number of advanced small- and medium-sized technologies.^7,12^

Given the plethora of technological configurations and treatment alternatives, the overall environmental impacts vary among systems. This depends on the type and quality of the wastewater treated, the specific nutrient removal approach (biological, membrane, physicochemical, hybrid, etc.), the use of chemicals, the energy and heat intensity, and whether or not the systems are tailored to resource recovery.^13^ Finding the optimal treatment approach requires identifying and balancing numerous trade-offs across contextual, operational, and supply-chain stages. Life cycle assessment (LCA) is a widely used environmental modeling methodology for evaluating and analyzing the life cycle impacts of products and technologies. LCA has been used extensively to assess environmental impacts and trade-offs of different WWM configurations.^14,15^

LCA application to wastewater has expanded over time, as reflected in the growing number and scope of literature reviews in this field. Previous reviews have focused on aspects such as the use of LCA in different technological configurations (for example, membrane systems or nutrient recycling configurations,^16–18^ sludge management,^19^ and state-of-the-art modeling^14,15,20^ ) or were focused on a specific geographical scope.^21,22^ However, a quantitative synthesis and harmonization of small and medium WWM (SM-WWM) systems are lacking, which prevents a clear understanding of their environmental impacts. In a context in which centralized systems are increasingly under pressure and higher degrees of pollutant removal and overall reduction of environmental impacts are required for all scales, identifying which systems and technologies could meet all required environmental criteria becomes relevant.

In this study, we present the first harmonization of LCAs for small and medium SM-WWMs. Life cycle inventories from peer-reviewed studies are synthesized and modeled using a harmonized framework. This way, fragmented data and modeling choices are harmonized and calculated under a consistent approach, thus enabling a fair comparison of SM-WWM. This study has three primary objectives: (i) to quantitatively assess environmental impacts and trade-offs across different technological approaches; (ii) to identify drivers of environmental impacts, and (iii) to provide a harmonized and transparent synthesis of existing inventories that enable meaningful interpretation across studies and highlight patterns. Our quantitative synthesis offers clear guidance for decision makers and supports the development of environmentally sustainable small- and medium-scale water treatment solutions for municipal settings. It also highlights key environmental hotspots and identifies potential improvements from both the treatment and modeling perspectives.

## 2 Methods

LCA harmonization is a quantitative approach that synthesizes and standardizes inventories from existing studies within a technological group.^23^ Introduced as a counterpart to meta-analysis, whose role in the LCA field has been debated, its aim is to reduce variability in methods, assumptions, and biases, enabling a consistent comparison of environmental impacts. While no harmonization has yet been conducted for SM-WWM, similar work exists for other technologies.^24^ In this sense, our study applies a framework aligned with previous harmonizations.^23,25^ The process comprised three steps: (1) screening and data extraction, (2) goal, scope, and inventory harmonization, and (3) LCA modeling and analysis. The harmonized inventories are included in the Supporting Information (SI).

### 2.1 Screening and Extraction

Data were obtained from existing life cycle inventories through bibliometric searches in the database Web of Science, following the PRISMA guidelines for systematic reviews.^26^ For the search string, we used (“Life cycle analysis” OR “LCA”) AND (“wastewater” OR “WWT” OR “WWM” OR “sewage”). This generalist search string was chosen because the concept of SM-WWM is not well-defined; it lacks a universally accepted size threshold and is not associated with a fixed set of technologies or treatment approaches. Thus, it was considered that targeted keyword searches could overlook suitable studies; instead, data collection was approached from a more comprehensive and broader approach, targeting as many studies as possible within the field of WWM. The search was carried out in January 2025. This yielded 1577 studies. Based on titles and abstracts, we identified 111 relevant peer-reviewed studies. An additional eight studies were included following a snowball approach, which led to a final shortlist of 119 studies.

From this shortlist, articles that respected the following criteria were retained (Figure 1):

- Perform an LCA and provide a transparent and replicable modeling framework.
- A maximum treatment capacity of 10,000 m^3^: This value was derived from a recent classification of treatment plants by size, which estimates that the highest treatment capacity for medium-scale systems is 47,000 people or 20,000 m^3^.^27^ Nonetheless, the typical European wastewater generation value is around 200 l per person, which means that 47,000 people would produce a volume closer to 10,000 m^3^.^28^
- Provide a harmonizable volumetric functional unit (FU), a system boundary or modeling flowchart, and unit-process data. In this context, harmonizable refers to a functional unit that can be converted to cubic meters.
- The LCI data must contain at least basic direct emissions to water and air, or it must be possible to calculate the full scope of key emissions based on provided data, including methane (CH_4_), carbon dioxide (CO_2_), nitrous oxides (N_2_O), chemical oxygen demand (COD), total nitrogen (and if possible other nitrogen compounds), and total phosphorus (and if possible other phosphate compounds). COD, biological oxygen demand (BOD), and suspended solids (SS) are currently not widely characterized, and their inclusion is not essential for the impact assessment.
- Provide the chemical composition of the influent (or at least an explicit description of the origin of the influent) and effluent (if absent, influent composition and treatment efficiency of the technology modeled). The influent must be exclusively from domestic sources.
- The physicochemical composition of the effluent must meet European standards for discharge, i.e., ∼125 mg/L COD, ∼25 mg/L BOD, and ∼35 mg/L TSS.^29^ Nonetheless, the systems do not need to be based in Europe, since the aim is to capture technologies and configurations deployed at a global level.

**Figure 1:**
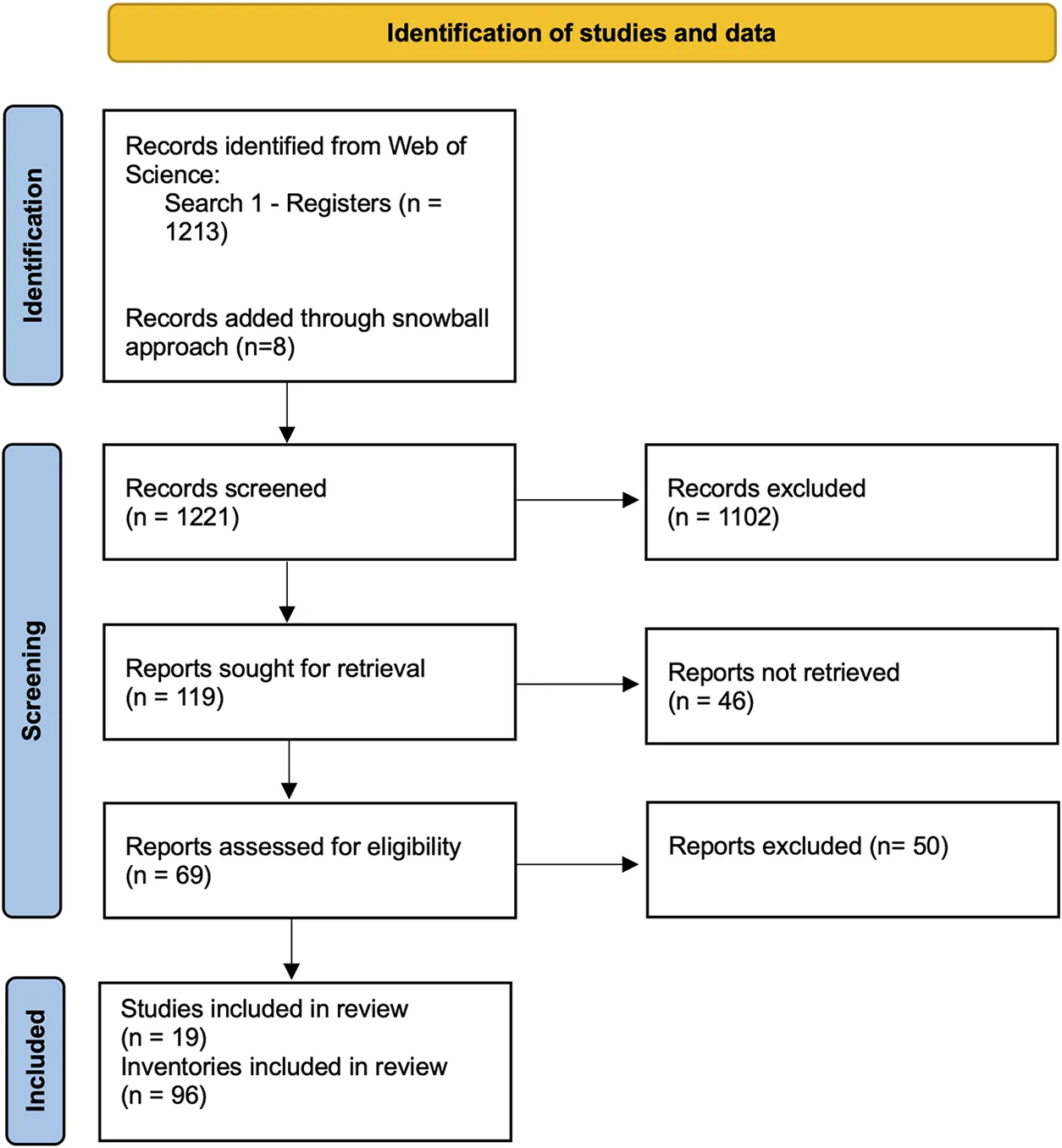
PRISMA flow diagram for systematic literature searches and study selection showing the sequential exclusion of studies.

Given the long technological lock-ins of wastewater treatment plants, often extending their operational lifetime over 30 years, it was considered that improvements at the plant level over time would be minimal.^15^ Thus, time was not considered part of the inclusion criteria as long as the system was deployed within 30 years of the study.

This resulted in 19 studies and 96 life cycle inventories (LCIs) (Table 2). The process flow that was followed to identify suitable studies is given in Figure 1, and an overview of the bibliometric results and rationale for inclusion or exclusion can be found in the SI. These LCIs were then clustered within broader WWM technology groups to facilitate cross-comparison and identify if a particular technological configuration displayed lower environmental impacts. The grouping was based on the technology undertaking the main nutrient and organic removal stages (mainly biological, physicochemical, or hybrid approaches). For example, if a conventional activated sludge system has constructed wetlands as a tertiary purifying step, then it would be classified under activated sludge.

Since the systems included depend on the aforementioned criteria, technology grouping is underpinned by data availability; thus, the resulting technology groups are not exhaustive of the potential options available for SM-WWM settings. A total of nine SM-WWM groups were defined according to the main technological configuration used to perform the treatment, which is at the secondary treatment (Table 1). A more detailed overview of these technologies can be found in SI-1. It should be noted that even though the inventories have been grouped within this classification, each system will have its unique process and there will be variations within each broader SM-WWM grouping. Finally, it is important to highlight that despite the use of broad search terms and a snowballing approach, relevant studies may have been missed due to inconsistent terminology used in the wastewater and LCA literature.

**Table 1:** Short Description of SM-WWM Technologies Considered in This Study along with Acronyms

**technology**	**acronym**	**description**	**removal system**
anaerobic membrane bioreactor	AnMBR	integrates the anaerobic digestion process with a membrane filtration step	biological/membrane
activated sludge (conventional)	AS	prevalent WWT that contains clumped masses of microorganisms present in the aeration tank, which break down the organic matter present in the wastewater	biological
bardenpho	BDP	this system is a modification of AS and consists of five distinct reactors which are anaerobic, anoxic, aerobic, anoxic, and aerobic; the process fosters nitrification–denitrification	biological
constructed wetlands	CW	constructed wetlands are engineered systems that replicate the natural processes found in wetlands, in a controlled environment; they treat wastewaters by a number of routes: filtration, adsorption, precipitation, ion exchange, plant uptake, and microbial degradation (both aerobic and anaerobic)	biological/natural filtration
membrane bioreactor (not anaerobic)	MBR	biological treatment combined with membrane filtration based on AS; the main difference with the AnMBR is that MBRs operate in aerobic conditions; generally, an aerobic bioreactor is followed by a membrane filtration tank, equipped with a return activated sludge (RAS) pipe	biological/membrane
modified Ludzack-Ettinger	MLE	the modified Ludzack–Ettinger is a multi-stage process that often combines an anoxic zone (where denitrifying bacteria are fostered) followed by an aerobic one (where the presence of added oxygen promotes the growth of nitrifiers). MLE is a modification of AS	biological
oxidation ditch	OD	oxidation ditches are variations of conventional activated sludge (AS), where a closed-loop pool system achieves simultaneous nitrification-denitrification by alternating aerobic and anoxic zones within the closed-loop system	biological
sequencing batch reactor	SBR	sequencing batch reactors are fill-and-draw, suspended-growth activated sludge systems. SBR carries out all AS functions within a single complete mix reactor by alternating operational processes (aeration, settling, decanting)	biological
upflow anaerobic sludge blanket	UASB	UASBs are usually formed by one reactor with a dense sludge bed at its bottom, through which the wastewater is pumped in an upflow direction; this results in an efficient mixing between biomass and wastewater, leading to rapid anaerobic decomposition	biological

### 2.2 Goal and Scope Harmonization

The goal of the harmonization is to eliminate modeling and inventory biases across SM-WWM systems, allowing for a fair and consistent comparison of the multiple treatment and management options available and highlight patterns and trends. In this sense, the harmonization aims to reduce methodological variability by standardizing key aspects such as system boundaries, substitution approaches, and inventory flows, but retains differences in operational, technical, and management decisions.

#### 2.2.1 Functional Unit

For this study, a volumetric FU is used, defined as treating 1 cubic meter of domestic wastewater with a discharge that meets European discharge limits (∼125 mg/L COD, ∼25 mg/L BOD, and ∼35 mg/L TSS, for nonsensitive areas).^29^ FUs in LCAs of SM-WWM are commonly defined either volumetrically (e.g., per m^3^ of treated wastewater) or in terms of person equivalent (PE).^18,21^ Previous research has concluded that it is preferred to use a PE FU in cases where either water use or water pollution varies considerably.^20^

Inventories that used liters or PE instead of cubic meters as the reference flow had to be harmonized. For PE, varying per-capita water use would limit comparability. Recent data suggest that the average European person produces around 200 L of wastewater per day,^28^ aligning closely with the 191 L/PE·day average across the included sources (Table 2). In consequence, a value of 200 L/PE·day was used to convert inventories based on PE into cubic meters.

**Table 2:** Studies Included for Harmonization and Summary of Parameters Provided in Each^a^

				emissions to water					emissions to air					
**study**	**SM-WWM**	**FU**	**L·PE**	**COD**	**BOD**	**SS**	**TP**	**TSS**	**N**_**2**_ **0**	**CO**_**2**_	**CH**_**4**_	**CM**	**MF**	**#**
Morelli et al. (2018)^30^	AS, MLE	m3	NA		Y	Y	Y		Y		Y	Y	^b^sub	2
Garfi et al. (2017)^31^	AS, CW	m3	200		Y	Y		Y	Y	Y	Y	Y	no	2
Li et al. (2024)^32^	CAS, AS	m3	NA	Y		Y	Y		Y	Y	Y		no	2
Foley et al. (2010)^33^	AS, MLE, OD, BDP,MBR, AnMBR	10^4^ m3	NA	Y			Y		Y		Y	Y	exp	47
Moussavi et al. (2021)^34^	SBR, OD, AS	m3	NA		Y		Y		Y		Y	Y	no	16
Vassalle et al. (2023)^35^	UASB	m3	NA	Y		Y	Y	Y	Y		Y	Y	^b^sub	3
Lijo et al. (2017)^36^	AnMBR, UASB	2000 PE.d	200	Y		Y	Y	Y	Y	Y	Y	Y	^b^sub	2
Lima et al. (2023)^37^	SBR	1 PE.yr	NA				Y		Y	Y	Y	Y	sub	1
Corbella et al. (2017)^38^	CW	m3	195		Y	Y			Y		Y	Y	^b^sub	3
Kalbar et al. (2013)^39^	AS	PE.yr	260				Y		Y	Y			no	1
Risch et al. (2021)^40^	CW	PE.d	100	Y		Y	Y		Y		Y	Y	^b^exp	2
Hospido et al. (2012)^41^	MBR	m3	NA	Y			Y		Y	Y	Y		sub	3
Pasciucco et al. (2023)^42^	AS	m3	NA	Y			Y		Y		Y		no	1
Lopsik (2013)^43^	AS	PE	NA	Y	Y	Y	Y	Y				Y	no	1
Estevez et al. (2022)^44^	AnMBR, MBR, AS	m3	NA	Y^b^			Y^b^	Y^b^	Y	Y	Y		^b^sub	2
Resende (2019)^45^	AS	m3	NA	Y		Y	Y		Y		Y	Y	no	1
Liu et al. (2018)^46^	OD	m3	NA	Y			Y	Y	Y	Y	Y		^b^sub	2
Kohlheb et al. (2020)^47^	SBR	m3	NA	Y	Y	Y	Y	Y	Y		Y	Y	no	1
Ortiz et al. (2007)^48^	AS, MBR	m3	227	Y	Y	Y	Y	Y	Y	Y			no	3

a SM-WMM = small and medium wastewater management. FU = functional unit. L·PE = liters per person equivalent, if the study is not using a volumetric FU. COD = chemical oxygen demand. BOD = biological oxygen demand. TN = total nitrogen. TP = total phosphorus. TSS = suspended solids. CO2 = carbon dioxide. CO = carbon monoxide. SO2 = sulfur dioxide. N2O = nitrous oxide. CH4 = methane. CM = construction materials. MF = multifunctionality. MF shows how multifunctionality was handled; Sub = substitution; Exp = expansion. # = number of inventories included in each study. AnMBR = anaerobic membrane bioreactor. UASB = upflow anaerobic sludge blanket. CW = constructed wetland. SBR = sequence batch reactor. AS = activated sludge. OD = oxidation ditch. MBR = membrane bioreactor. MLE = modified Ludzack–Ettinger. BDP = Bardenpho.

b Means that it was not specified in the text but was inferred by the authors by assessing how avoided products were modeled.

Since all inventories are calculated using the same FU (1 cubic meter), variations in plant capacities are not explicitly modeled or differentiated and thus not captured in the current study. Given the linear nature of the LCA calculations, these differences do not influence the results.

#### 2.2.2 System Boundary and Multifunctionality

The system boundary (Figure 2) describes the processes and life cycle stages that are included in the LCAs. This study covers the wastewater treatment process from the inlet of wastewater to the system through its discharge of cleaned water into receiving water bodies. Given the many combinations of the systems included in the study, the treatment trains in the system boundary have been simplified to visualize the general composition of the SM-WWM systems instead of depicting an accurate representation. In some of the systems modeled, energy and fertilizer were recovered during the treatment stages. This was not the case for all of the systems included, since only a few had processes such as struvite recovery or biogas capturing. Resource recovery options were retained as originally modeled in the source studies. Furthermore, sludge was assumed to be sent to an incineration plant with heat and energy recovery, which is a common handling process in Europe. In these cases, multifunctionality is handled using substitution, which is often used in wastewater treatment LCA studies.^49–51^ Nevertheless, a sensitivity analysis regarding this choice is conducted by applying economic allocation to the systems that displayed the highest recovery of resources, since this methodological choice can have a large influence on results and interpretation (see SI-9).

**Figure 2:**
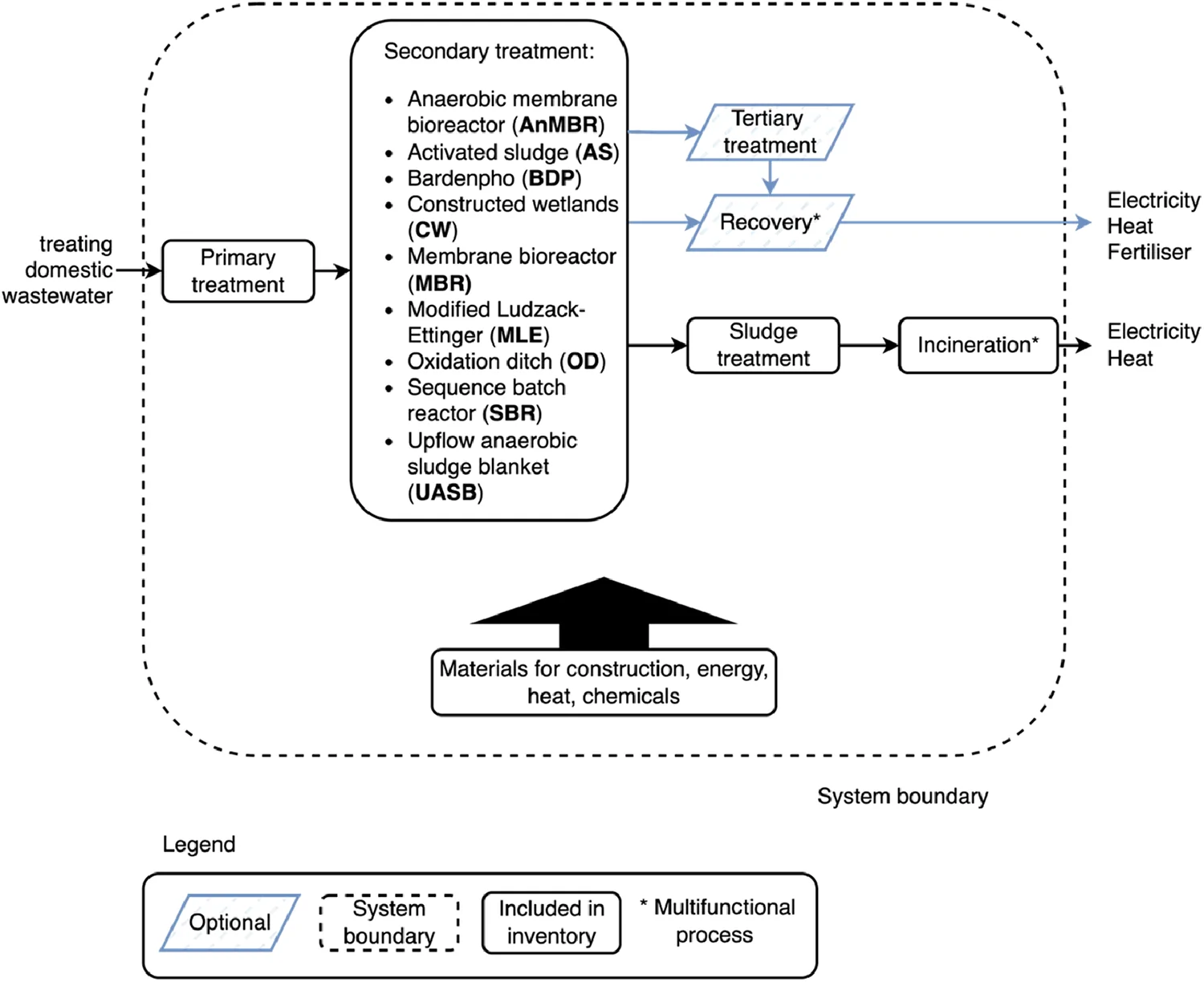
System boundary, which includes construction, operational inputs, direct emissions to air and water, primary, secondary, and tertiary treatment if applicable, and recovery of resources if relevant. Secondary treatment refers to the wastewater management alternatives included in this study. The boundary of the study includes the upstream and downstream impacts of producing the required inputs for the wastewater treatment. Application and use of recovered products are excluded. The reference flow is 1 m^3^ of incoming domestic wastewater. A list of the specific technologies modelled can be seen in the SI in the inventories of each system and in SI-8.

### 2.3 Inventory Harmonization

#### 2.3.1 Construction, Infrastructure, Chemical Use, and Sludge Handling

Construction is often omitted in WWM LCAs on the premise that its impacts are negligible compared to operation (Table 2). However, this assumption may not hold for small-scale systems, where construction impacts normalized per unit of treated water can be relevant. Thus, where missing, construction was incorporated using previous work by Foley and Doka,^33,52^ who have been acknowledged as an accurate approximation of real-plant inventory data for systems with biological nutrient removal (BNR).^53^ For harmonizing technology groups that don’t conform with standard BNR infrastructure, an estimation was done using available inventories from the included studies within each technology category.

The specific technological configurations of the systems that are design choices are not harmonized. For example, some plants have additional steps for struvite precipitation, but this is not a common design choice across the systems included in this study. In these cases, we do not redesign all other plants to incorporate struvite precipitation since this is a design choice that falls outside of the scope of the study. Sludge treatment was harmonized by assuming that incineration. This limits potential fertilizer recovery, but since this practice is not allowed in numerous countries, incineration is a more transferable choice. A detailed overview of chemical use and sludge handling is available in SI2-4.

#### 2.3.2 Emissions to Air

Inventories where greenhouse gas emissions (GHG) were included were kept as reported. However, many studies omit several GHG sources (see Table 2). Often, CO_2_ is assumed to be entirely biogenic, particularly in studies based on the first edition of the IPCC guidelines (pre-2019). More recent research has shown that wastewater has a non-biogenic carbon content ranging from 4 to 28%, with many of the estimates pointing toward a median of 15%, and is released during organic matter oxidation in treatment processes.^54–57^ Consequently, 15% of the carbon content was attributed to fossil sources with the remaining 85% considered biogenic.

The remaining emissions, such as CH_4_ and N_2_O, were estimated based on organic and nutrient removal efficiency, using either the updated IPCC guidelines (2019) or emission factors from Wang et al.^58^ This study provides emission factors differentiated by technology, allowing tailored estimations for specific SM-WWMs. Additionally, their factors cover multiple greenhouse gases (GHGs), enabling proxy estimations of CO_2_ emissions, where COD or CO_2_ data are unavailable. However, harmonization using these factors should be approached cautiously as many values derive from Chinese contexts and may not be fully representative elsewhere. An in-depth overview of GHG harmonization can be found in SI-6 and SI-8.

#### 2.3.3 Emissions to Water

Some studies did not report all necessary emissions to water (Table 1), but they could be calculated using provided information. To estimate this, we first assumed an influent ratio of COD, TN, and TP of 100:17:3, based on BOD/TN/TP concentrations of 200/35/5,6 for medium-strength wastewater and 400/69/11 for high strength.^13^ Subsequently, we used technology-specific removal efficiencies from the literature to estimate the remaining TN and TP emissions. When this was not possible, we used point-source data for nutrient removal from WWTPs in European wastewater.^59^ More information is available in SI-5 and SI-8.

### 2.4 LCA Modeling and Analysis

The impact assessment was done using ReCiPe Midpoint 2016,^60^ as it provides a comprehensive set of environmental impact categories and has been previously identified as the most appropriate for wastewater-related impacts.^20,21^ We focused specifically on marine and freshwater eutrophication and climate change, as these are often viewed as the main impact categories concerning WWT.^20^ Toxicity categories are also key when assessing the impact of WWT. However, because of the lack of necessary inventory data, such as heavy metal and micropollutant concentrations in wastewater, this impact category was omitted from our study. We also calculated correlations between energy and chemical use and nitrogen and phosphorus removal. We carried out the analysis in Brightway 2.0, with the ecoinvent-3.9.1-cutoff database, and performed attributional LCAs for all systems modeled. Primary data calculations are available in SI2-5, and an overview of how each system was harmonized is included in SI8 and the inventory excel file is provided in the SI. The LCA results for all of the categories characterized under the ReCiPe impact assessment are found in SI9. The python scripts are available in the GitHub repository and the SI.

## 3 Results

### 3.1 Direct Emissions to Air Are Key Contributors to Climate Change

Overall, direct emissions, energy use, and chemical use are the main contributors to the climate change impacts. As can be seen in Table 2, only 8 out of the 19 studies included reported all three key GHG emissions, and most did not account for fossil CO_2_, meaning that these emissions are likely under-reported and inconsistently treated in previous studies. According to the results, direct emissions dominate 70% of the systems, particularly those with lower energy and chemical input. However, in 42% of the cases, the sum of operational inputs (energy and chemicals) outweighs the direct air emissions. Construction and sludge disposal generally contribute less to climate change impacts (Figure 4). UASBs present the highest climate change impacts (2.22 kg CO_2_-equiv per FU on average, Figure 3), and these are predominantly driven by direct emissions to air. These emissions are, however, partly due to the open-system design of the tertiary treatments coupled to the UASBs such as polishing ponds (UASB2). Within UASBs, the system with the highest emissions is the one followed by a sequencing batch reactor (SBR) and a trickling filter using food waste as an alternative carbon source. This system has a wide scope of direct and fugitive emissions from each treatment train and composting unit. It should be noted that this inventory is one of the most complete in terms of direct-emission coverage. The other technologies with the highest emissions are variations of the activated-sludge system. In contrast, these emissions are driven by comparatively higher use of concrete during construction (AS1 in Figure 4) and high employment of aluminum chloride and polyacrylamide (AS2 and AS12 in Figure 4).

**Figure 3:**
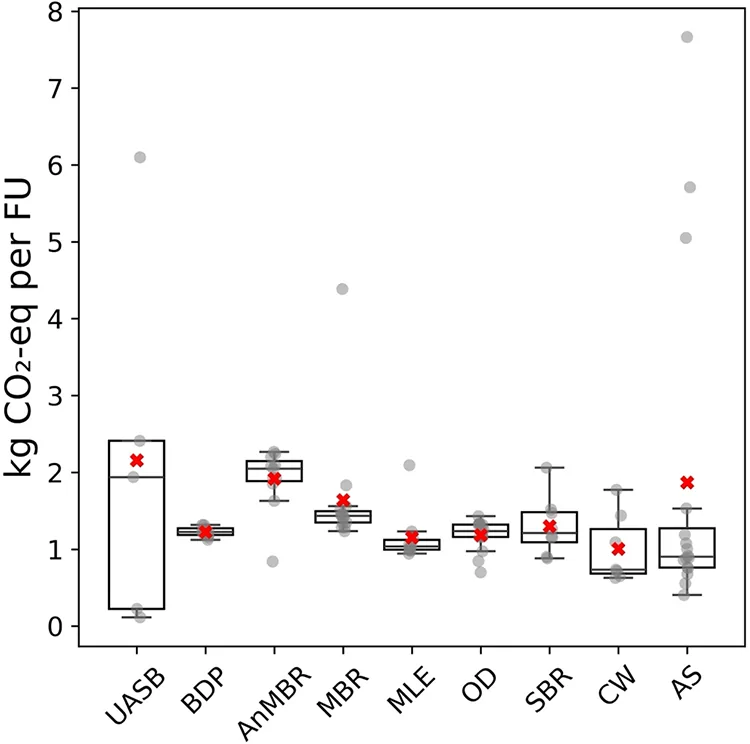
Life cycle impact assessment results for climate change per functional unit (m^3^ of wastewater treated). Each gray dot represents a specific technology configuration within a wastewater management system (e.g., UASB shows five individual data points corresponding to five separate LCAs of systems using UASB as the main treatment technology). Boxplots indicate the interquartile range; whiskers represent the 95% confidence interval. The red crosses represent the technology’s mean impact value. AnMBR = anaerobic membrane bioreactor; UASB = upflow anaerobic sludge blanket; CW = constructed wetland; SBR = sequence batch reactor; AS = activated sludge; OD = oxidation ditch; MBR = membrane bioreactor; MLE = modified Ludzack–Ettinger; and BDP = Bardenpho.

**Figure 4:**
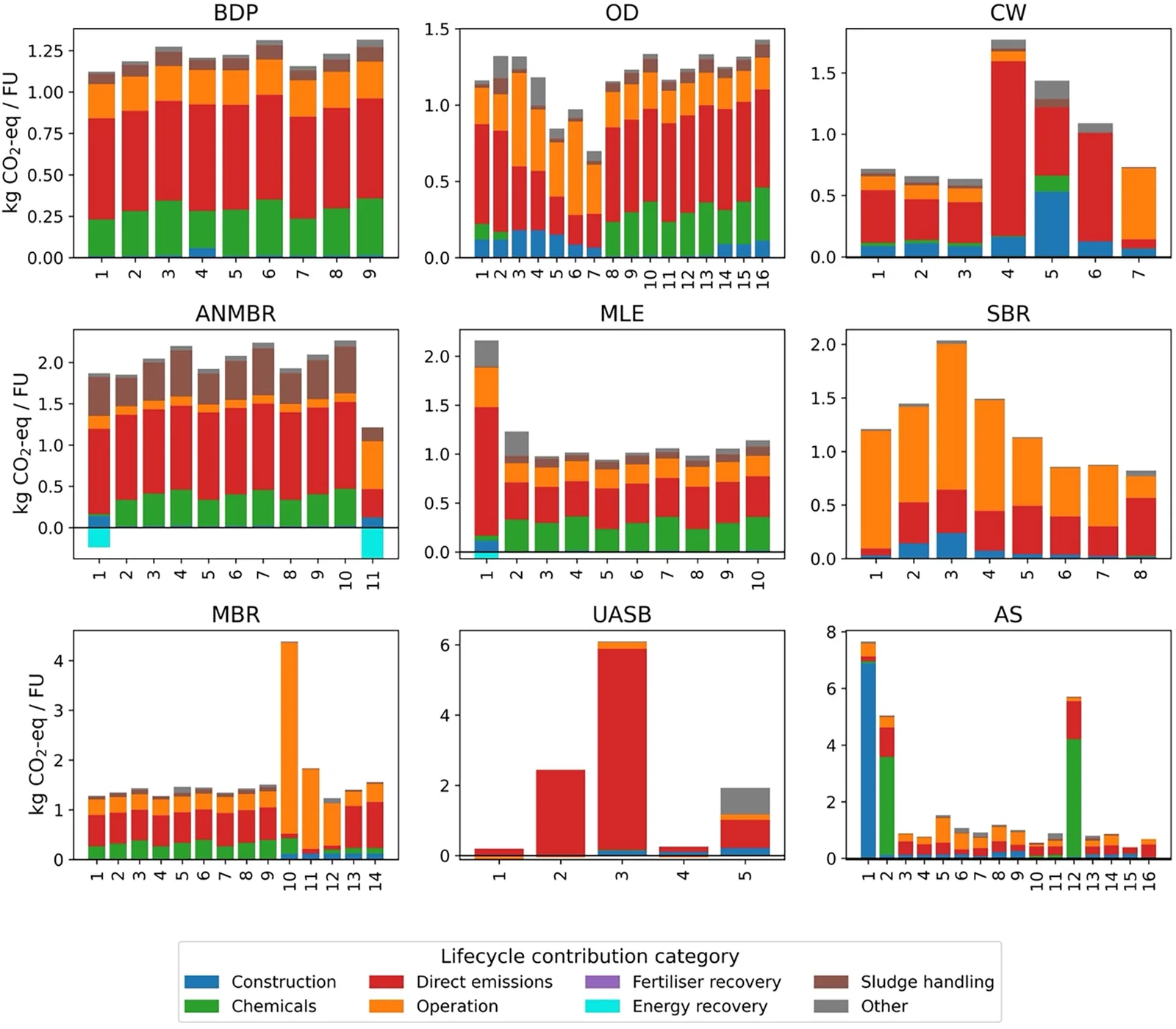
Contribution analysis to climate change impacts, grouped by processes and life cycle contribution categories. Recovery is plotted below zero and is already deducted from the total impact. The processes and flows included in each grouping are available in the SI and python repository. The numbers on the *x*-axis represent each individual configuration within the technology group and correspond to the activity code of the technology’s inventory, also available in the inventory excel file in the SI (e.g.,: Bar 1 in AnMBR corresponds to AnMBR1 in the AnMBR inventory tab in the SI).

CWs result in the lowest climate change impacts, producing on average 0.97 kg of CO_2_-equiv per FU (Figure 3). This was expected, as CWs often avoid high energy and chemical uses. Although direct emissions are the main driver of climate change, the relative contribution of construction and sludge disposal is higher in these cases, highlighting the need to include these stages in WWM LCAs (Figure 4).

Energy recovery is considered an effective way to mitigate energy usage and reduce the operational climate change impacts. The system with the highest energy recovery is SBR1, which produced energy through a heat pump installed in the effluent. The other systems with high energy recovery are two AnMBR1 and AnMBR11 (see SI8). The first upgrades biogas through a trickling filter, while the second separates black and gray water and recovers biogas from the BW through a combined heat and power conversion unit (Figure 4). Fertilizer recovery plays a minimal role in reducing the climate change impacts.

Looking at the contribution analysis, it is noticeable that sludge handling plays a considerably higher role in the climate change impacts of SBR8 when compared with the other systems in the category. This is explained by the fact that SBR8 includes measured data on sludge produced during the treatment, whereas these data are missing in inventories SBR1–SBR7. For the latter cases, proxy calculations and assumptions based on average values were used to include sludge handling in the LCA, resulting in considerably lower impacts. Although this could be specific to this system, it raises the importance of including all relevant stages in the inventory to ensure accurate results.

Since the benefits of resource recovery are often underpinned by the way in which multifunctionality is handled, we performed a sensitivity analysis regarding this choice by conducting economic allocation on two systems with high resource recovery (AnMBR1 and SBR1; SI-10). Substitution yields either a more conservative estimate or one that is comparable to economic allocation, showing that the environmental impacts resulting from this study are not overly sensitive to the choice regarding multifunctionality handling.

### 3.2 Sequencing Batch Reactors Cause the Least Eutrophication

The lowest eutrophication impacts are observed in the SBR systems (Figure 5). This is in part driven by a specific SBR configuration (inventory SBR1, see the SI), which is also the WWTP with the lowest total eutrophication impact due to its high effectiveness at reducing N and P emissions (Figure 6). This SBR system separates blackwater (BW) and gray water (GW) treatment, with the SBR treating the former. The treatment train includes a nanofiltration and ozonation following the SBR, which, combined with the fact that GW usually has lower pollutant concentrations than combined (GW+BW) or BW alone, results in a low eutrophication impact. The BW is treated in a UASB, with the sludge undergoing pasteurization and dewatering and the liquid undergoing struvite precipitation and ammonia stripping. These processes use a set of different chemicals that result in the recovery of NPK pellets. Ultimately, this means that only the effluent from the GW is discharged, which reduces eutrophication impacts. It should be noted that the SBRs included in this study come from three sources; however, seven out of the nine are from Moussavi et al. (2021), thus dominating the inventories in this category.

**Figure 5:**
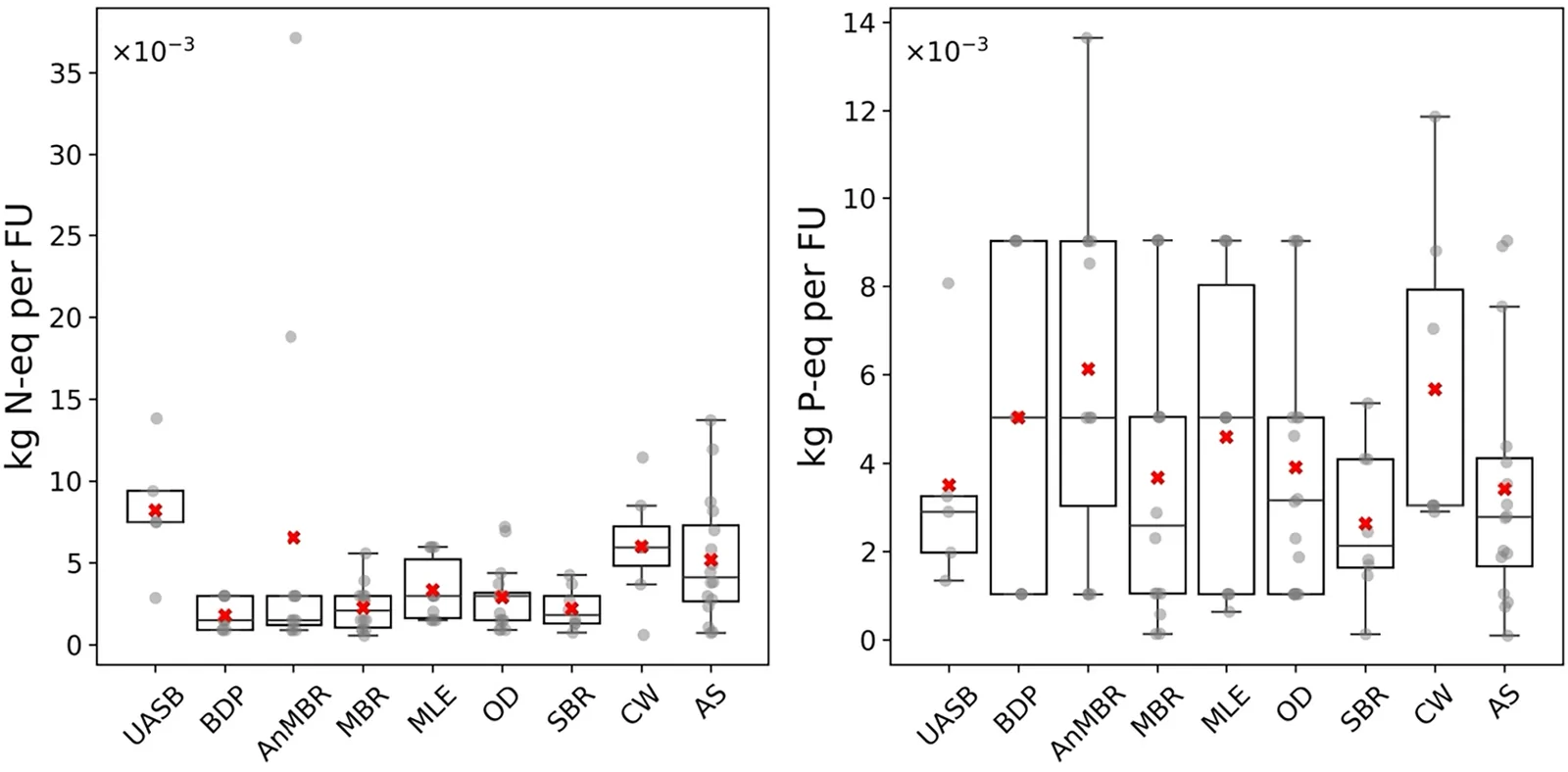
Life cycle impact assessment results for freshwater eutrophication and marine eutrophication. Each dot represents a specific technology configuration within a wastewater management system (e.g., UASB shows five individual data points corresponding to five separate LCAs of systems using UASB as the main treatment technology). Boxplots indicate the interquartile range; whiskers represent the 95% confidence interval. The red crosses represent the technology’s mean impact value.

**Figure 6:**
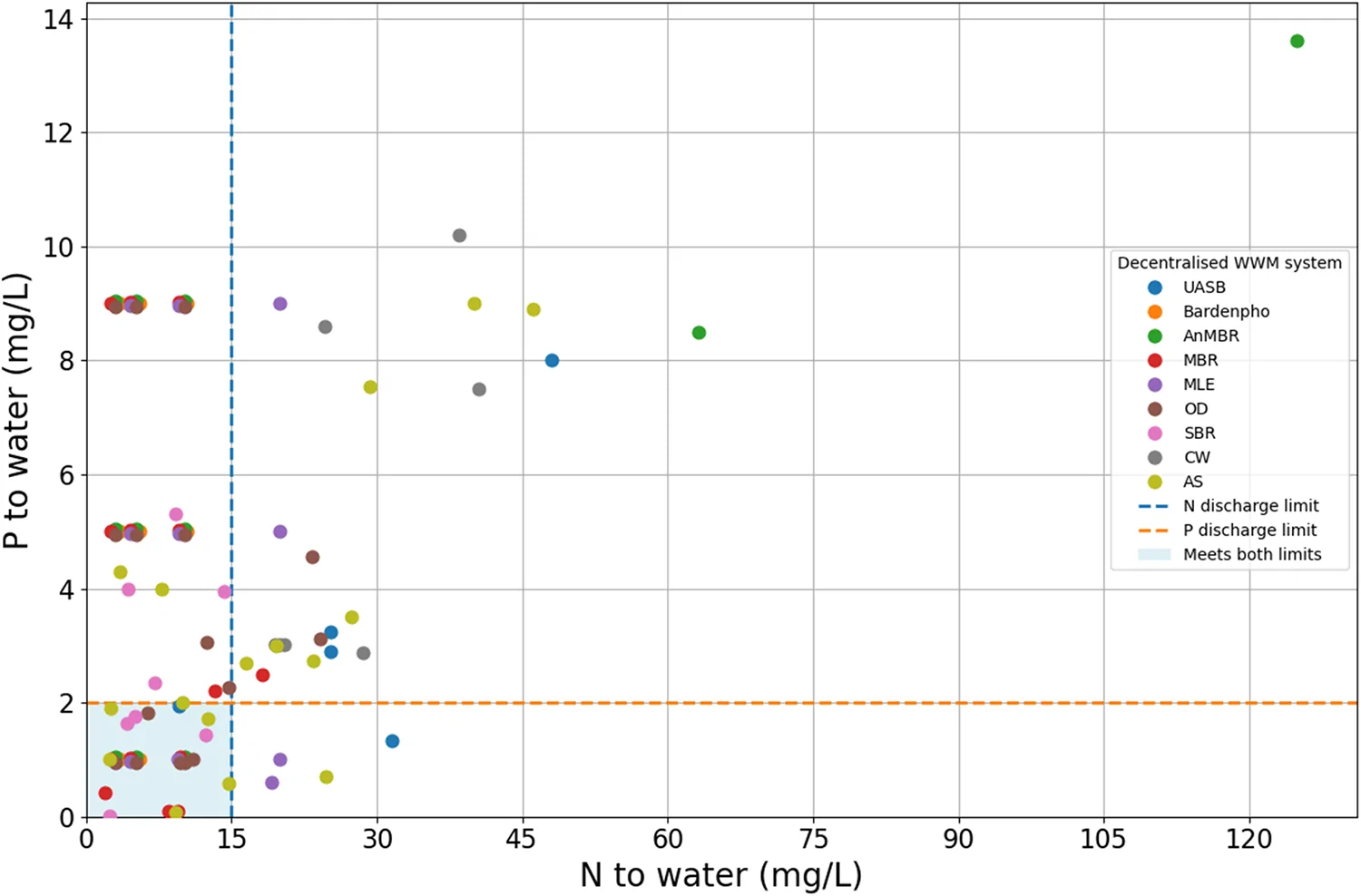
Direct P and N emissions for each system. The orange and blue bars represent the nutrient discharge limit for sensitive areas, provided in milligrams per liter (mg/L). The blue shade showcases the overlap between both discharge limits.

In addition to SBR1, two other systems showed among the lowest eutrophication impacts: a hollow-fiber MBR with both anoxic and aerobic segments (MBR) and a conventional activated sludge (CAS) system with phosphorus/nitrogen recovery (Figure 6). Although the MBR does not recover nutrients, these technologies generally demonstrate a high organic and nutrient removal capacity. In the MBR system, anoxic tanks provide an oxygen-deprived environment that promotes the growth of denitrifying bacteria, facilitating nitrogen reduction, while aerobic zones support organic matter oxidation.^61^ Additional organic and nutrient removal is undertaken at the filtration stage.^62^ The combination of mechanical and biological removal systems results in a high effluent quality. The CAS system also combines mechanical pretreatment with a primary biological treatment stage and is specifically designed for nutrient recovery. This is achieved by dosing iron chloride and polyacrylamide (PAM), promoting high coagulation and flocculation of small particles, including phosphates and suspended solids.^63^ This results in the recovery of resources and reduction of the amount of pollutants in the effluent.

Several of the SM-WWM systems included in the study can reduce N and P discharges to a threshold compatible with sensitive areas (15 mg of N/L, 2 mg of P/L; Figure 6). Interestingly, the systems included in this study appear to be more effective at limiting nitrogen than phosphorus in the effluent, as 71% meet a discharge limit of 15 mg of N/L, but only 35% limit phosphorus below 2 mg of P/L. The systems modeled show little statistical difference in nitrogen reduction and show an arguably consistent discharge, which would fall within the ranges of varying physicochemical composition of the influent, whereas phosphorus removal displays higher variations in results (Figure 5).

Finally, given the influence of operational inputs into nutrient reduction, we carried out a correlation analysis to explore the direct influence of increasing chemical and energy use on eutrophication impact reduction. Energy consumption increases do not translate into eutrophication reductions (Figure 7). On the other hand, chemical dosing displays a higher, albeit moderate, relationship with the reduction of eutrophication. This is more noticeable in the reduction of phosphorus emissions, suggesting a higher dependence on chemical dosing to limit freshwater eutrophication impact.

**Figure 7:**
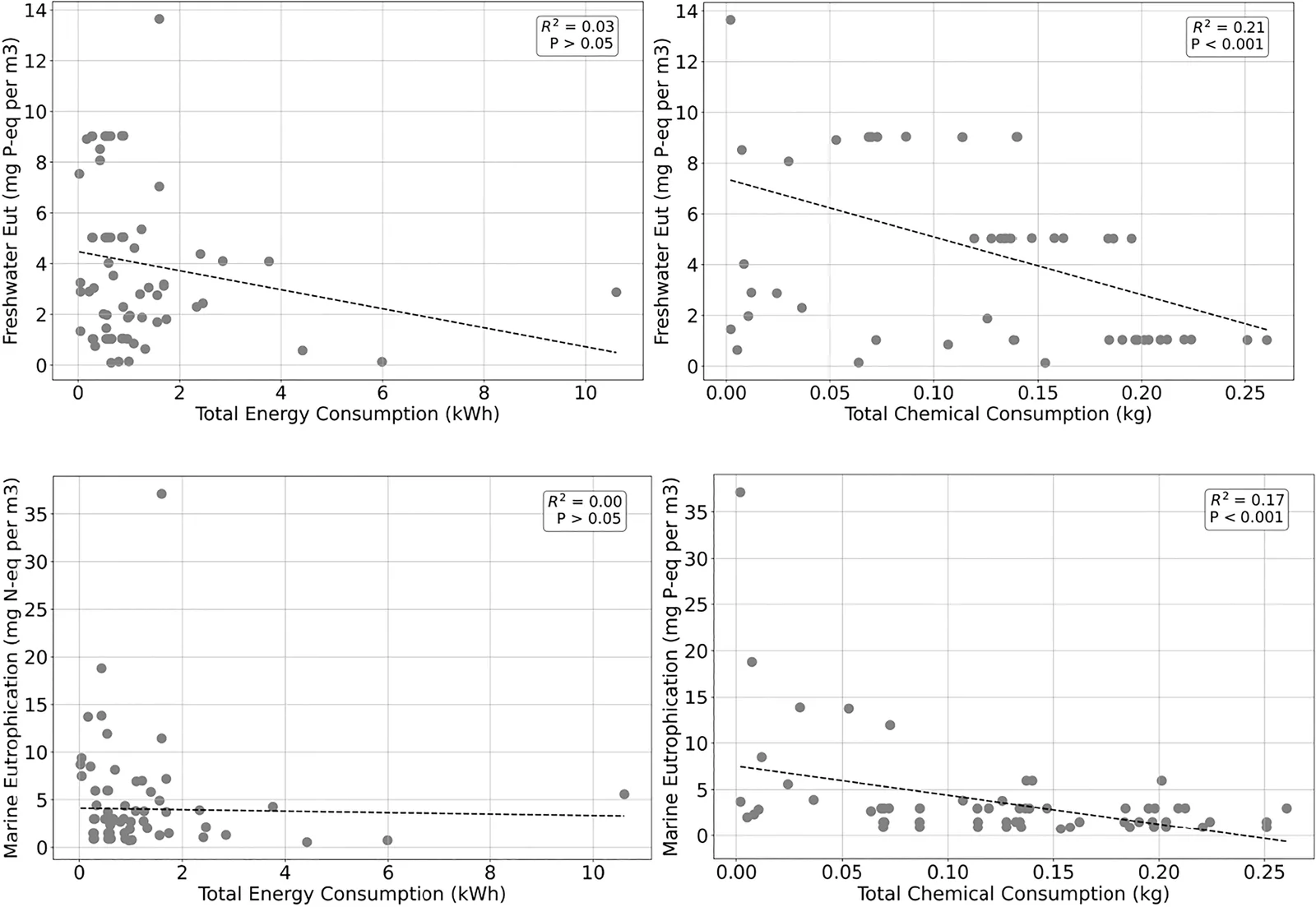
Correlation analysis between energy, chemical use, and freshwater (top panel) and marine (bottom panel) eutrophication reduction. The influence of increasing energy or chemical use on eutrophication reduction was assessed through linear regression. The chemicals included in the analysis are polyacrylamide, chlorides such as polyaluminum chloride and ferric chloride, potassium carbonate, sodium hypochlorite, and phosphoric acid.

## 4 Discussion

### 4.1 Decarbonizing Small- and Medium-Scale Systems Requires Reducing Direct Air Emissions

Direct air emissions from SM-WWM systems remain the key contributors to climate change impacts. The primary gases emitted are N_2_O, CH_4_, and CO_2_, alongside other molecular forms, such as ammonia (NH_3_) and carbon monoxide (CO). N_2_O is emitted during different stages of biological nitrogen removal, mainly nitrification and denitrification, incomplete heterotrophic denitrification, and hydroxylamine oxidation.^55,64^ Although recent advances have enhanced the understanding of autotrophic and heterotrophic microbial performance, these emissions cannot be entirely avoided in nitrogen removal processes.^65^ CH_4_ emissions can occur at different stages of the WWM system: at the sewage line before reaching treatment, during the treatment process, as dissolved CH_4_ in the effluent, from leakages in the plant, and during sludge handling.^58,66^ Similar to N_2_O, there are pathways to reduce the amount of CH_4_ emitted, most notably through its recovery in the form of biogas. CO_2_ emissions are primarily linked to the degradation of dissolved organic carbon in the influent wastewater, which is one of the main water pollutants.^67^ While a portion of this organic carbon is stabilized in sludge, the remainder is oxidized and emitted as CO_2_ and related compounds, mostly from short-lived biogenic carbon sources but also from fossil carbon.^67,68^

Abating these direct GHG emissions requires novel approaches to the microbial and metabolic performances of biological wastewater processes or devising innovative means of capturing these emissions, which are both complex and lengthy solutions. Furthermore, recent research highlighted that a large percentage of GHG emissions in WWTPs come in the form of fugitive CH_4_ emissions during anaerobic digestion, raising considerations about the overall benefits of this type of emission capture and utilization systems.^69^

This underscores a critical shift in the emission reduction priorities. While grid decarbonization and energy-saving innovations will continue to reduce operational emissions, their impact may be marginal compared with the mitigation potential of reducing direct GHG emissions. From a modeling perspective, this is also a factor to consider in the case of including grids with lower integration of renewable energy sources, since the climate change impacts from energy consumption may dominate those from the direct emissions (see SI-11). As such, we encourage efforts to be made on further understanding and optimizing biological pollutant removal, prioritizing systems that result in lower direct GHG emissions.

While direct emissions dominate the climate change impact category, it is possible that the contribution of sludge management and disposal is being downplayed. First, actual production of sludge appears to be larger than the average that was used in this study to carry out the harmonization, as seen in the inventories for which measured data were available. Second, the impacts from sludge management are underpinned by the choice of disposal and the total distance associated with it.^70^ Although assessing the impacts of different sludge disposal approaches is not within the scope of this paper, previous research has highlighted that this choice can result in noticeable variations in climate change impacts.^71–73^ In this sense, it is first encouraged to understand these results in the context of sludge incineration. Second, it is advised for future studies to calculate and include an estimated amount of sludge produced, to better understand what the contribution of this life cycle stage to the overall system is.

### 4.2 Limiting Eutrophication whilst Reducing Externalities Could Be Achieved with SBRs

Our study shows that numerous modeled systems release an effluent that meets both N and P discharge limits (around 35%). However, nitrogen reduction is more commonly achieved across all systems, with 71% meeting N discharge standards, compared to only 35% that comply with P discharge limits (Figure 6). Although these discharge limits are applicable to sensitive areas, they serve as a tangible basis for comparison. One potential explanation is that nitrogen is commonly reduced using biological treatments, which are well-established and often more suitable for decentralized and medium-sized systems.^74,75^ Phosphorus emissions, although they can also be limited using biological processes, are often reduced using chemical dosing or membrane filtration.^50,76^ This is visible in our study, as it appears that chemical dosing has a higher influence on reducing P-based emission (Figure 7), further supporting the hypothesis that to reduce P-based emissions from SM-WWM, a higher reliance on chemicals may be needed. Since both of these options result in more costly processes and operational complexities, their suitability for SM-WWM is reduced.^77^ Another explanation behind this is that smaller systems (<1000 PE) are often exempt from stricter discharge directives.^78^ Given that some of the systems included in this study are designed to operate below this capacity threshold, controlling nutrient release would be beyond the scope of these technologies, thus resulting in higher P and N release.

In this regard, lower P removal and recovery raise two important considerations. First, lowering P emissions in SM-WWM systems reduces freshwater eutrophication and could help improve freshwater quality.^79^ For instance, a recent study explored the correlation between numerous parameters of wastewater management and ongoing freshwater ecosystem damage in Europe and found that most of the discharge to freshwater streams is from WWTPs with a size PE <10,000.^8^ If the systems tailored to these sizes (mostly medium-scale WWM) indeed have lower ability to reduce P emissions, then this could be one contributing factor to the ongoing poor ecological status in European freshwater bodies. Furthermore, this highlights a key area for the improvement of SM-WWM systems. Second, there are concerns around the global availability of virgin phosphate, a nonrenewable resource, and the economic feasibility of its extraction.^80,81^ Limiting phosphorus emissions through recovery could reduce the reliance on virgin minerals and help shorten supply chains for P-based fertilizer use in the agricultural sector. Nonetheless, environmental feasibility studies are required to understand better potential trade-offs and implications of these systems, such as increased reliance on chemicals, environmental impacts of applying recovered fertilizer and sludge, or the need for complex and expensive membrane technologies.^76,82–84^ The financial viability of recovered P is also contested, given the often small quantities in which P can be recovered.

As presented in the results, the SBRs included in the study show low marine and freshwater eutrophication while maintaining minimal chemical dosing. A particular strength of SBRs appears to be a higher efficiency in reducing P emissions compared to the rest of the treatment systems. In SBRs, the cyclical anaerobic/anoxic system fosters the growth of denitrifying phosphorus bacteria, and the accumulated solids get effectively settled as part of the batch system.^85,86^ Furthermore, one of the main advantages of SBRs is that the batch system does not require separate tanks for the different biological processes due to the specific granules undertaking the pollutant removal, leading to a reduced area requirement for the infrastructure.^87,88^ The lower reliance on chemicals, high nutrient removal capacity, and comparatively lower land use make SBRs a suitable system for small- and medium-scale sizes. In addition to this, even though SBRs display the third highest mean climate change impacts, these predominantly come from energy use, which can be effectively mitigated with higher renewable energy grid penetration.

It is important to highlight that, despite the good nutrient reduction capacity of SBRs, these systems often had comparatively higher COD/BOD emissions into the water compartment. Nonetheless, organic pollutants are not characterized in the ReCiPe impact families. In this regard, we identify an ongoing gap around the characterization of organic pollutants and their contribution to water quality-related impacts. Other impact methodologies characterize COD, such as CML-IE.^89^ However, they do this by combining all nutrient and organic pollutants into one broader eutrophication impact category, resulting in a less nuanced assessment of the impacts.^89^ Likewise, suspended solids are currently not characterized by any impact methodology and are also sources of eutrophication and toxicity. In this sense, the impact on aquatic ecosystems can only be partially assessed.

### 4.3 Limitations, Research Gaps, and Opportunities

We identify several methodological limitations of this synthesis. First, determining what falls under medium-sized capacity is a challenge, since this is not defined nor agreed upon. While this type of classification may not be relevant to the design and delivery of WWM, it can be a challenge when undertaking research that requires some form of classification based on treatment capacity. Although the choice made in this study is contrasted with other scientific sources, it is possible that this synthesis has missed inventories that would be effectively considered as SM-WWM. Moreover, given the linear nature of LCA calculations, the influence of size and capacity is not captured, even though the study includes inventories that represent varying treatment capacities. In this sense, the analysis is done assuming that all systems modelled fulfil the same role, whereas in reality, not all of the systems modelled would be deployable across all capacity requirements of SM-WWM.

Second, a key challenge during harmonization of SM-WWM is that the physicochemical properties of the influent and effluent cannot be equalized; thus, the treatment capacity for each configuration is not harmonized and depends on the composition at the inlet. To minimize this, we set a control variable for all influents to come exclusively from domestic sources. Nonetheless, despite being similar, the influent varies from study to study. Adjusting the influent would require either for all studies to provide treatment efficiency (or both influent and effluent, which is not the case) or to perform process models of each configuration, which is beyond the scope of this article.

Third, the fact that organic pollutants are not characterized by the ReCiPe impact assessment method means that it is not possible to have a complete pollution profile of these systems. Furthermore, the lack of characterization factors and data regarding new pollutants, such as PFAS and pharmaceuticals, alongside the general absence of data on heavy metal transfer in wastewater, results in an incomplete ecotoxicological assessment.

Overall, the main limitation is that a high number of studies had to be excluded from the harmonization for not meeting inventory transparency and transferability criteria. Thus, the statistical robustness of the results is underpinned by the small sample size in some of the technologies, the dominance by a small number of studies, and the technologies available from the data selection. As such, the results are intended to highlight systematic patterns and dominant drivers rather than definitive performance differences. On the basis of this, we encourage LCA practitioners going forward to ensure their studies can be effectively replicated. In this study, a baseline for data and modeling completeness and transparency is provided.

## 5 Conclusions

This study presents the first effort to conduct LCA synthesis and harmonization of small- and medium-scale WWM systems. We compared environmental impacts across nine treatment technologies and several configurations and operational approaches. While our approach is limited by data availability, it offers a basis for the assessment of prominent medium-scale WWM approaches. We encourage LCA practitioners focusing on WWM to provide a similar level of detail during inventory analysis and ensure that data are accessible, transparent, and transferable. Future studies would benefit from clearly reporting influent and effluent characteristics, emission calculation methods, background database versions, and complete life-cycle inventories to support reproducibility and synthesis.

Given current data limitations, the value of this study lies in identifying dominant impact drivers and data gaps rather than establishing statistically significant performance differences among technologies. Our study shows that direct GHG emissions during the treatment process consistently contribute the most to climate change impacts. While increasing energy efficiency is important from an economic perspective, addressing direct emissions is likely to considerably reduce the climate change impacts of WWM. We also observe a notable contribution to climate change from chemical dosing for nutrient precipitation and sludge conditioning.

On the basis of the results of this study, SBRs are arguably more suitable in addressing current and emerging sustainability challenges for WWM at the small and medium scale. Although there are numerous technologies that show lower average climate change impacts compared with those of SBRs, the impacts from SBRs seem to be associated with energy use, which would be reduced as the share of renewable energy increases. The SBRs in this study consistently showed low eutrophication impacts, combining low GHG emissions with a high nutrient removal efficiency. Furthermore, one of the key advantages of SBRs is that they can significantly reduce land use due to their compact plant design, making them suitable for urban, peri-urban, and densely populated areas.

## Supplementary Material











## Notes

Life cycle inventories for all the systems modelled (XLSX)
Folder containing the GitHub repository of Jupyter notebooks explaining the functions and logic used in the modelling and calculations (ZIP)
LCA results for 16 impact categories according to ReCiPe 2016 Midpoint (XLS)
Results from the keyword search and selection of inventories (XLSX)
Overview of calculations for the flows and processes included in the inventories, overview and summary of harmonisation for each system,detailed description of SM-WWM systems included, and full LCA results (PDF)
