# Supplementary material for: Global Harmonization of Life Cycle Assessments for Small- and Medium-Scale Wastewater Treatment
Source: ACS ES T Water. 2026 Oct 1;6(10):6725–38. doi: 10.1021/acsestwater.6c00580 (PMC13660387; doi:10.1021/acsestwater.6c00580)
Supplement: Supplementary file 1 [file ew6c00580_si_005.pdf]

Supplementary Information for "A global harmonization of life cycle assessments for small and medium-scale wastewater treatment"

Emilio Solis[1*], Nils Thonemann[1], Ranran Wang[1, 2], Arnold Tukker[1, 2, 3], Valerio Barbarossa[1, 4]

[1] Leiden University, Institute of Environmental Sciences (CML), Leiden, the Netherlands

[2] Nanjing University, Institute for the Environment and Health, Suzhou, China

[3] Netherlands Organization for Applied Scientific Research TNO, 2595 DA Den Haag, the Netherlands

[4] PBL Netherlands Environmental Assessment Agency, Den Haag, the Netherlands

Classified as Internal | Intern

Table of Contents

S1. SMALL AND MEDIUM SCALE WASTEWATER MANAGEMENT SYSTEMS (MWWM) OVERVIEW 3

S2. CONSTRUCTION PROCESSES 7

S3. CHEMICAL DOSING 8

S4. SLUDGE PRODUCTION 8

S5. EMISSIONS TO WATER 11

S6. EMISSIONS TO AIR 12

S7. LINEAR RELATIONSHIP BETWEEN CHEMICAL, ENERGY AND EUTROPHICATION 15

8. HARMONISATION OVERVIEW 16

S9. LCA RESULTS 35

S10. SENSITIVITY ANALYSIS USING ECONOMIC ALLOCATION TO HANDLE MULTIFUNCTIONALITY 37

S11. ELECTRICITY MIX SENSITIVITY ANALYSIS 44

REFERENCES 47

Classified as Internal | Intern

S1. Small and medium scale wastewater management systems (mWWM) overview

Table S1. Small and medium scale wastewater management systems overview

mWWM	Description	Removal system	Advantages	Disadvantages
AnMBR	Integrates anaerobic digestion process with a membrane filtration step.	Biological/ Membrane	• High organic removal<br>• Often effective at eliminating micropollutants and pathogens<br>• Low sludge production	• Low nutrient removal as a standalone<br>• Membrane fouling
AS	Prevalent WWT that contains clumped masses of microorganism present in the aeration tank, which break down the organic matter present in the wastewater.	Biological	• One of the best-known systems<br>• Low installation cost	• Cell mass formed during the biological processes becomes part of the sludge<br>• High operation, since it needs mechanical aeration and often required chemical dosing
BDP	This system consists of five distinct reactors which are: anaerobic, anoxic, aerobic, anoxic, and aerobic. The process fosters nitrification-denitrification.	Biological	• High organic and nutrient removal	• Complex operational parameters<br>• Requires four/five separate reactor tanks<br>• Knowledge gaps about optimal HRT and attached biomass
CW	Constructed wetlands are engineered systems that replicate the natural processes found in wetlands, in a	Biological/ Natural filtration	• Low operational costs<br>• Efficient organic and nutrient removal rates<br>• Depending on the fate of the	• High land occupation, which means that they are mostly suited as tertiary or purification steps<br>• Nutrient removal is sensitive to

Classified as Internal | Intern

	controlled environment. They treat wastewaters by a number of routes; filtration, adsorption, precipitation, ion exchange, plant uptake, and microbial degradation (both aerobic and anaerobic).		biomass, it can help in carbon sequestration<br>• Can provide broader ecosystemic functions	temperature variations
MBR	Biological treatment combined with membrane filtration. The main difference with the AnMBR is that MBRs operate in aerobic conditions. Generally, an aerobic bioreactor is followed by a membrane filtration tank, equipped with a return activated sludge (RAS) pipe.	Biological/ Membrane	• Can be a compact systems<br>• High organic and nutrient removal<br>• Can remove challenging pollutants in the filtration step<br>• Produces less sludge compared to other biological systems, partly due to the RAS system and partly due to the membrane filtration	• High energy use<br>• Complex operational parameters, including membrane fouling
MLE	The Modified Ludzack-Ettinger is a multi-stage process that often combines an anoxic zone (where denitrifying bacteria are	Biological	• Simpler than other nitrogen removal technologies	• Although designed for biological nitrogen removal, it has low P-removal, and has lower N-removal than other systems such as

	fostered) followed by an aerobic one (where the presence of added oxygen promotes the growth of nitrifiers).			BDP (although easier to operate)
OD	Oxidation ditches are variations of conventional activated sludge (AS), where a closed-loop pool system achieves simultaneous nitrification-denitrification by alternating aerobic and anoxic zones within the closed-loop system.	Biological	• Effective nitrogen and organic pollutant removal rates<br>• Compact operation, requiring only one pool<br>• Produces highly stable sludge	• As a standalone, not effective at eliminating phosphorus<br>• As other processes based on AS systems, it's energy intensive<br>• Produces a lot of sludge, which required large infrastructure to handle it
SBR	Sequence batch reactors are continuous-flow, suspended-growth activated sludge systems. SBR carries out all AS functions within a single complete mix reactor by alternating operational processes (aeration, settling, decanting).	Biological	• Compact operation, requiring only one basin<br>• Continuous flow<br>• Simultaneous nitrification-denitrification, providing efficient nitrogen removal<br>• Although they require mechanical aeration, SBRs are more energy-efficient than other biological processes	• As other systems based on AS processes, it's prone to produce large amounts of sludge<br>• Most favourable operation requires optimal balancing of organic and nutrient load – high maintenance and oversight needed

			• Can achieve simultaneous high-rate removal N and P	
UASB	UASBs are usually formed by one reactor with a dense sludge bed at its bottom, through which the wastewater is pumped in an upflow direction. This results in an efficient mixing between biomass and wastewater, leading to rapid anaerobic decomposition.	Biological	• High organic removal rate<br>• Low sludge production<br>• It’s compact, and suitable as effective primary treatment in a more complex treatment train<br>• High production of biogas as a result of the anaerobic degradation of organic matter<br>• Operate effectively with low retention times	• On its own, it has low nutrient removal rates<br>• If not captured, the biogas produced can be a significant contributor to the climate change impacts of the system<br>• It’s sensitive to changes in pollutant load, as the sludge granules are disturbed in sudden alterations in pH and the physicochemical properties of the influent<br>• Susceptible to solids in the influent, requiring a thorough primary stage

References used for the description of the Small and medium scale wastewater management systems:

AnMBR: Kanafin et al., (2021), Osman & Hodaifa, (2023)

AS: Rezai & Allahkarami, (2021), Srivastava & Chattopadhyay, (2021)

BDP: Banayan Esfahani et al., (2018), Ashrafi et al., (2019)

CW: Ebie et al., (2013), Yan & Xu, (2014), Stanbury et al., (2017)

MBR: C. Wang et al., (2021), Y. Wang & Serventi, (2019), Kitanou et al., (2021), Cheng et al., (2022)

MLE: S.R. Nikou et al., (2025)

OD: Guo et al., (2013), Hartley, (2008), Xie et al., (2011), Luo et al., (2020)

SBR: Dutta & Sarkar, (2015), Irvine et al., (1989), Tantak et al., (2014)

Classified as Internal | Intern

UASB: Rattier et al., (2022), Mainardis et al., (2020), Ahmed et al., (2015)

S2. Construction processes

For construction, there are three main archetypes, to the author's best knowledge: Doka (2003), Foley et al (2010) and Morera et al (2020). In applying the equations generated by Morera et al (2020), for which the variables are supposed to be included as capacity in $m^3 \cdot d^{-1}$, the results obtained appear to reflect a disproportionality large value. For example, for a plant with a capacity of 2500 $m^3 \cdot d^{-1}$, the concrete for the secondary treatment would be 27,452,000 Kg, using equation (1) below:

$$Concrete_{kg} = 4.27\mathrm{E}^{1} \cdot \mathcal{X} - 2.41\mathrm{E}^{4}$$

Equation 1

Adjusting to a reference flow of one cubic meter of water and converting kilograms to cubic meters of cement (assuming an industry standard volume to weight ratio of 1:2200), this results in 4.99 m^3/m^3. This is approximately 18,700 times more than the 2.66E-04 cubic meters of concrete needed for a similar-sized plant per cubic meter of wastewater, according to Doka (2003) and Foley et al (2010). It's probable that this value is referring to the concrete needed for the entire lifetime of the plant, instead of the reference flow of $m^3 \cdot d^{-1}$. In this case, a further correction would be needed, including the estimated operational lifetime of the plant. Since there is no further clarification of this in the text, it was decided to use the factors developed by Doka (2003), since they seem to provide accurate enough estimations (Morera et al, 2020).

Regarding construction, where inventories did not specify material processes, the following were selected:

- For unspecified steel and metals, "steel, chromium steel 18/8" (18% chromium 8% nickel), which makes it resistant to corrosion and oxidation and suitable to water installations.
- For unspecified concrete, a generic flow "concrete, all types to generic market for concrete, normal strength" located in Switzerland [CH].
- For unspecified bitumen, "bitumen seal, VA4", a standard bituminous cover.
- For unspecified copper, "copper cake" was used as proxy, as it was assumed that most copper used in the installation will be for electrical systems.

Classified as Internal | Intern

Most inventories include some type of plastic consumption, usually polyvinyl chloride (PVC), polyethylene (PE) and polypropylene (PP). In the cases in which this is specified to be for the piping system (either for the treatment infrastructure or for the sewage system), a more accurate estimation can be made, since there are flows specific for plastic pipes for wastewater management.

Transport for construction materials is also often included, but the type is unspecified. In these cases, we have assumed the transport to be “transport, freight, lorry 3.5-7.5 metric ton, EURO6”. This was the typical transport modelled in the inventories that did specify the type of transport. In the cases where transport was missing, estimates from Doka (2003) were utilised.

S3. Chemical dosing

Polyelectrolytes and polymers are often used for WWT in coagulation, flocculation, and dewatering of sludge, and are often part of removing suspended solids (Bolto & Gregory, 2007; Saravanan et al., 2022). Many inventories include polyelectrolytes, but this process is not included in Ecoinvent, and in none of the inventories it’s specified how polyelectrolytes are modelled. Although there are different compounds that can be used as polyelectrolytes in wastewater treatment, polyacrylamide (PAM) is a commonly used one, and thus was selected to include the background processes of polyelectrolyte and polymer use (Abbasi Moud, 2022; Bolto & Gregory, 2007; Chen et al., 2024; Huang et al., 2016).

Calcium hydroxide is included in some inventories. Generally, this is used to raise the alkalinity and control the pH of the sludge mixture to stabilise it (Czechowski & Marcinkowski, 2006). Calcium hydroxide is not a flow included in inventory databases, so potassium hydroxide, which is also utilised as a pH adjusting agent in WWT (Z. Li et al., 2018), is used as a proxy.

S4. Sludge production

The sludge line is a key aspect of wastewater treatment. Some inventories excluded the sludge treatment and handling, either because they assumed the same fate within their study, or because it was argued that it would add negligible impacts to the overall system (for example, Corbella et al., 2017). In most cases, sludge was transported to agricultural fields and applied as biosolids. In these cases, given the content of P/N in digested and stabilised sludge, production of synthetic fertiliser was assumed to be avoided. This practice is however not permitted in all countries, and there are concerns about potential toxicological impacts of sludge application in agricultural fields. In all studies besides Foley et al., (2010), biosolids application was modelled using substitution instead of

system expansion, which can overlook important trade-offs such as an increase in eutrophication and toxicology. Thus, modelling biosolids application as organic fertiliser without incorporating the downstream impacts of this practice can lead to biases in the results. Coupled with the fact that this practice is either uncommon or prohibited in many countries, for this study we assumed incineration of sludge with energy recovery, which is another common way of handling sludge (Scrinzi et al., 2023). For this, we used the Ecoinvent process “treatment of sewage sludge, 70% water, WWT, WW, average, municipal incineration”, which includes upstream and downstream technosphere and biosphere flows.

Unless otherwise stated, it was assumed that the incineration facility operated independently of the wastewater treatment plant (WWTP), necessitating the inclusion of transport processes. An average transport distance of 100 km between a WWTP and a municipal incineration plant was considered. The same value has been used in other studies, although this will likely vary considerably depending on the proximity to an incineration plant (Högstrand et al., 2024). Consequently, this assumption will likely be lower than actual values for many countries with centralized or no incineration plants; however, it remains within the range of values reported in other studies (Domini et al., 2022; Seiple et al., 2017; Tarpani et al., 2020).

In cases where sludge production data were reported in the original studies, the same quantity was assumed to be directed to the incineration plant. When sludge was applied as biosolids, the amount of sludge generated was estimated either based on the conversion rate associated with fertilizer substitution or derived from transport data, typically expressed in tonne-kilometres.

In the event in which the amount of sludge produced per reference flow is not specified, there are number of possible ways to estimate this. In its most simple form, sludge yield is often calculated based on relationship of volatile solids (MLVSS) and chemical oxygen demand (COD) (Fida et al, 2021):

$$Y = \frac{gMLVSS}{gCOD}$$

Equation 2

Where Y is sludge yield, expressed by the volatile suspended solids by the substrate consumed measured in COD. In reality, more variables play a role in dictating the true amount of sludge produced, such as loading and hydraulic retention time, volume of

Classified as Internal | Intern

reactor or tank and decay rate, although less studies will include this type of design variables. When this data is available, there are numerous equations available to calculate sludge yield or obtain conversion efficiencies.

For instance, for many biological treatments, sludge production rate (P_{Xv}) can be obtained by:

$$P_x = \frac{YQ(S_o - S_e)}{1 + \theta_c K_d}$$

Equation 3

Where Y is the yield constant (expressed as the relationship between mass of cells and mass of substrate in milligrams); Q is the flow; S_o and S_e are the influent and effluent organic content; θ_d is the sludge retention time; and K_d is the decay time. Since LCA studies rarely provide such detailed design data, conversion efficiency equations (which are variations of equation 1) are more suitable to calculate sludge yield. In these cases, there are two options: the COD-MLSS conversion efficiency or the SS-MLSS conversion efficiency (Kong et al., 2021). The former is calculated using removed COD amount with growth of MLSS, and is expressed as:

$$\Upsilon COD = \frac{V_r \Delta MLSS_t}{Q \cdot t \cdot COD_r}\%$$

Equation 4

Where V_r is the volume of the reactor: ΔMLSS is the accumulation of MLSS over a time period t; Q is the flow rate; and COD_r is the removed COD.

The latter is calculated using the relationship between SS and MLSS, and is expressed as:

$$\Upsilon SS = \frac{V_r \Delta MLSS_t}{Q \cdot t \cdot SS_r}\%$$

Equation 5

Classified as Internal | Intern

Where the only different is that yield is calculated based on the removal of total SS, as opposed to COD.

When possible, a conversion factor was calculated using equations 1, 3 and 4. Nonetheless, studies often include aggregated SS, instead of a breakdown of the category to show which portion of the organic matter is volatile. According to different sources, the ratio of MLVSS to TSS usually ranges between 0.6 and 0.8 (Sun et al, 2024; Jiang et al, 2025), thus a value of 0.7 MLVSS/tSS was used.

If no parameters were available, a value was estimated using European averages. Sludge production varies per country (depending for example of proportion of population connected to treatment lines, type of water and sludge treatment, and occupational habits), but numerous sources provide a range between 15 and 25 Kg/PE.year (Di Giacomo & Romano, 2022; Kelessidis & Stasinakis, 2012). Assuming an average wastewater production of 200 L/PE.day in Europe (Jones et al., 2021), this results in a range of 0.20-0.34 Kg of sludge per cubic meter, which is around 40g PE.d.

In some of the LCIs, energy is recovered through anaerobic digestion of the sludge, which can either take place inside or outside of the plant. In the cases in which the sludge is processes inside the plant but sent to an outside facility to further treatment and recovery, this was only included in the boundary if the original inventory provided input processes. For example, Morelli et al (2018) include sludge disposal into a landfill that has high methane capture during anaerobic digestion which leads to high rates of energy recovery; however, no additional inputs are provided, and their inventory includes a considerable negative flow for energy recovery. In our assessment, this is an incomplete flow, and given the lack of additional information about this anaerobic digestion process, this unit process is replaced be an average European value from Ecoinvent. In the cases in which all necessary inputs (such as additional energy or chemicals required for an upgraded anaerobic digestion process) are provided, these are left as part of the boundary without the need for replacing them with Ecoinvent processes, such as in Lima et al (2023).

S5. Emissions to water

In some cases, emissions to water had to be estimated performing calculations based on the data provided. Firstly, removal efficiencies were calculated based on averages from studies within the same technology and similar treatment trains. For organic removal, in the case that either COD or BOD were reported, but not both, a ratio of 2.4 COD:BOD was assumed, and an assumption was made that similar rates of organic removals would apply to both organic parameters. For the cases in which N and P was not reported, values provided in Metcalf and Eddy et al., (2014) about typical composition of domestic

Classified as Internal | Intern

wastewater were used to estimate ratios (found in Table 3-18, p. 221). In this sense, a BOD:TN:TP ratio of ~100:17:3 was established (based on BOD/TN/TP concentrations of 200/35/5,6 for medium strength wastewater; 400/69/11 for high strength).

Alternatively, IPCC factors were used inversely to estimate emissions to water based on emissions to air. When missing, removal efficiencies were estimated using values form literature or from available inventories with similar technologies and treatment trains. Since only three studies did not report removal efficiency of nutrient pollutants categorised under SBR, AS and CW, it was only necessary to estimate removal efficiencies under these technologies:

- SBR: COD ~85% (Khan et al., 2024; Kitanou et al., 2021; Kulikowska et al., 2007)
- AS: COD ~ 87% (Zhang et al., 2023); TN ~ 68%; TP ~ 76% [calculated as the average from removal efficiencies found in Li et al (2024) (TN~78%, TP~92%), Morelli et al (2018) (TN~45.6%, TP~91%) and Pasciucco et al (2023) (TN~81%, TP~45%)]
- CW: TN~30%; TP~40% (Cui et al., 2016)
- Average biological/mechanical nutrient removal: TN ~ 55%; TP ~ 65% (Pistocchi et al., 2023)

S6. Emissions to air

To estimate emissions to air, the updated 2019 IPCC guidelines for wastewater treatment were used. These often work by applying an emission factor to organic or nutrient removal quotients.

For N_2O emissions to air, an emission factor (EF) is applied to the amount of influent N, as seen below in Table S2. For CH_4 emissions, a similar approach is taken, but the EF for the specific treatment is configured using methane correction factors (MCF) prior to applying the EF. This is done using equation 6:

$$EF_j = B_o \cdot MCF_j$$

Equation 6

Where the EF_j is the product of the maximum CH_4 producing capacity (B_o) and the methane correction factor for the specific treatment (MCF_j). The specific MCFs can be seen in Table S3. Default B_o is 0,6 CH_4/kg BOD and 0.25 kg CH_4/kg COD.

In cases in which the IPCC factors were not applicable to a specific inventory, factors from Wang et al (2022) were used. This was specifically the case to calculate CO_2 emissions, for which the IPCC guidelines are not specific enough. Whereas the updated report provides some guidance to calculate fossil carbon in wastewater, the reference units provided in the

Classified as Internal | Intern

report are not often included in inventories. For example, one of the provided carbon ratios is that 14% of particulate organic carbon (POC) is estimated to be of fossil origin. Nonetheless, organic compounds are often reported together and is seldom separated into particulate carbon, dissolved carbon, etc. The most straightforward factor is provided by Yoshida et al., (2014), who estimated that around 14(±3)% of influent carbon is of fossil origin. Thus, to estimate fossil CO_2 we aimed to apply a factor to influent total carbon when reported, which was seldom the case. As a second option, the factors developed by Wang et al were used. Nonetheless, these factors are used to calculate all carbon emissions, not only those of fossil origin. As such, after obtaining the CO_2 emissions using factors from Wang et al, we applied the fossil ratio provided in the updated IPCC guidelines (2019).

It's worth noting that the factors found in Wang et al (2022) are developed for the Chinese context, and may not be fully representative in broader applications (mainly due to organic and nutrient load variations across regions), and emissions estimated with these factors should be treated with caution. However, since these factors are exclusively calculated for domestic wastewater, and given variations in physicochemical compositions of influent is not too dissimilar across regions, the percentages proposed should represent a reasonable estimate for other contexts.

Classified as Internal | Intern

Table S2. Emissions factor for N2O emissions, from updated IPCC guidelines (2019)

TABLE 6A.5 (NEW)
N_2O EMISSION FACTORS IN FULL-SCALE DOMESTIC WASTEWATER TREATMENT PLANTS

Type of treatment process[1]	Categories	References	N_2O emission factor (kg N_2O-N/kg N)
AO	Biological Nitrogen Removal (BNR)	Daelman *et al.* (2015)	0.028
AO	BNR	Foley *et al.* (2010)	0.021
AO	BNR	Foley *et al.* (2010)	0.045
A2O	BNR	Foley *et al.* (2010)	0.013
SBR	BNR	Foley *et al.* (2010)	0.023
OD	BNR	Foley *et al.* (2010)	0.0080
IA	BNR	Kimochi *et al.* (1998)	0.0005
EA	BNR	Foley *et al.* (2010)	0.015
A2O	BNR	Wang *et al.* (2016)	0.013
CAS	BNR	Aboobakar *et al.* (2013)	0.00036
AO	BNR	Rodriguez-Caballero *et al.* (2014)	0.00116
OD	BNR	Masuda *et al.* (2018)	0.00016
AO	BNR	Masuda *et al.* (2018)	0.0013
AO	BNR	Masuda *et al.* (2018)	0.0049
Separate-stage BNR	BNR	Ahn *et al.* (2010)	0.00019
Bardenpho	BNR	Ahn *et al.* (2010)	0.0036
Step-feed BNR	BNR	Ahn *et al.* (2010)	0.011
MLE	BNR	Ahn *et al.* (2010)	0.0007
MLE	BNR	Ahn *et al.* (2010)	0.0006
OD	BNR	Ahn *et al.* (2010)	0.0003
Step-feed BNR	BNR	Ahn *et al.* (2010)	0.015
Step feed, plug flow	BNR	Ni *et al.* (2015); Pan *et al.* (2016)	0.019
SBR	BNR	Bao *et al.* (2016)	0.029
SBR	BNR	Rodriguez-Caballero *et al.* (2015)	0.038
Plug flow	Non-BNR	Ahn *et al.* (2010)	0.0041
Plug flow	Non-BNR	Ahn *et al.* (2010)	0.0036
Step-feed non-BNR	Non-BNR	Ahn *et al.* (2010)	0.010
Plug flow	Non-BNR	Masuda *et al.* (2015)	0.023
AO	Non-BNR	Bao *et al.* (2016)	0.013
IA	Non-BNR	de Mello *et al.* (2013)	0.0016

[1] AO; Anaerobic-oxic activated sludge process, A2O; Anaerobic-anoxic-oxic activated sludge process, SBR; Sequencing batch reactor, OD; Oxidation ditch, IA; Intermittent aeration process, EA; Extended aeration process, CAS; Conventional activated sludge process, MLE; Modified Ludzack-Ettinger.

Classified as Internal | Intern

Table 1. Methane correction factors (MCFs) used to obtain specific CH4 EFs. From updated IPCC guidelines (2019)

TABLE 6A.2 (NEW)
MCFS BASED ON MEASURED CH_4 IN FULL-SCALE DOMESTIC WASTEWATER TREATMENT PLANTS

Type of treatment process (Name of plant)	Reference	MCF[1]
Activated sludge (Stickney)	Bellucci *et al.* (2010)	0.017
Activated sludge (Northside)	Bellucci *et al.* (2010)	0.004
Activated sludge (Egan)	Bellucci *et al.* (2010)	0.014
Activated sludge	Czepiel *et al.* (1993)	0.013
Biological nutrient removal (Kralingseveer)	Daelman *et al.* (2013)	0.03
Biological nutrient removal (Kortenoord)	Daelman *et al.* (2013)	0.02
Biological nutrient removal (Papendrecht)	Daelman *et al.* (2013)	0.04
Sequencing batch reactor (Holbæk)	Delre *et al.* (2017)	0.038
Activated sludge (Källby)	Delre *et al.* (2017)	0.048
Biological nutrient removal (Lundtofte)	Delre *et al.* (2017)	0.014
Biological nutrient removal (Lynetten)	Delre *et al.* (2017)	0.015
Activated sludge	Kozak *et al.* (2009)	0.09
5-stage Bardenpho	Kyung *et al.* (2015)	0.07
Anaerobic/anoxic/oxic (A2O) process	Wang *et al.* (2011)	0.003

[1] MCF shown was calculated from data presented in studies, including measured CH_4 emissions from wastewater treatment plant (not including sludge digesters), influent organics in wastewater, estimates of organics removed in sludge, and the IPCC default B_o value.

S7. Linear relationship between chemical, energy and eutrophication

A simple linear regression (least squares fit) was used to estimate the influence of higher chemical or energy use in the removal of N and P-based nutrients. The function for the linear regression is:

$$\gamma = \beta_1 \chi + \beta_0$$

Equation 7

Where γ is the nutrient release (kg N-eq or kg P-eq); χ is the chemical (kg) or energy (kWh) consumption; β_1 is the slope; and β_0 is the intercept.
To compute R^2, the following equation is used:

$$R^2 = 1 - \frac{\Sigma\, (\gamma_i - \hat{y}_i)^2}{\Sigma\, (\gamma_i - \bar{y}_i)^2}$$

Equation 8

Where γ_i is the observed eutrophication value; $\hat{y}_i$ is the value predicted by Equation 5; $\bar{y}_i$ is the average eutrophication value across systems; $\Sigma\, (\gamma_i - \hat{y}_i)^2$ compiles residual sum of squares; $\Sigma\, (\gamma_i - \bar{y}_i)^2$ compiles the total sum of squares. The python script for this calculation is in the repository.

Classified as Internal | Intern

8. Harmonisation overview

Treatment system (code)	Configuration	Harmonisation overview	Source
Activated sludge (AS1)	AS with secondary settler and disinfection unit. Sludge treated in centrifuge.	Emissions to air calculated using IPCC factors, emissions to water calculated using influent ratios based on influent BOD and removal efficiency for standard AS processes.	Garfi et al 2017
Activated sludge (AS2)	Standard AS with anaerobic digestion	Fossil carbon calculated assuming a ratio of BOD:COD of 2,4, then using Wang et al (2022) factor for carbon dioxide in AS, then applying a factor of 0,15 to estimate fossil carbon, as per IPCC. Construction from Doka (2003). Sludge production based on European averages.	Morelli et al 2018
Activated sludge (AS3)	Standard AS process	Construction from Doka (2003)	Li et al (2024)
Activated sludge (AS4)	Vegetation-AS process, with anaerobic-anoxic-oxic stages	Construction from Doka (2003)	Li et al (2024)
Activated sludge (AS5)	Return activated sludge process, with clarification and disinfection	COD Calculated using IPCC factors for CH4, which for a AS is 7.5gCH4/KgCOD (inverse calculation based on the methane emissions calculated in the study). Assuming an ~85% COD reduction, which is an average reduction for AS. Carbon emissions calculated using Wang et al 2022, based on COD removed. sludge production based on European averages.	Moussavi et al 2024

Classified as Internal | Intern

Activated sludge (AS6)	Return activated sludge process, with clarification and disinfection	COD Calculated using IPCC factors for CH4, which for a AS is 7.5gCH4/KgCOD (inverse calculation based on the methane emissions calculated in the study). Assuming an ~85% COD reduction, which is an average reduction for AS. Carbon emissions caculated using Wang et al 2022, based on COD removed. Sludeg production based on European averages.	Moussavi et al 2024
Activated sludge (AS7)	Return activated sludge process, with clarification and disinfection	COD Calculated using IPCC factors for CH4, which for a AS is 7.5gCH4/KgCOD (inverse calculation based on the methane emissions calculated in the study). Assuming an ~85% COD reduction, which is an average reduction for AS. Carbon emissions calculated using Wang et al 2022, based on COD removed. Sludge production based on European averages.	Moussavi et al 2024
Activated sludge (AS8)	Return activated sludge process, with clarification and disinfection	COD Calculated using IPCC factors for CH4, which for a AS is 7.5gCH4/KgCOD (inverse calculation based on the methane emissions calculated in the study). Assuming an ~85% COD reduction, which is an average reduction for AS. Carbon emissions calculated using Wang et al 2022, based on COD removed. Sludge production based on European averages.	Moussavi et al 2024

Activated sludge (AS9)	Return activated sludge process, with clarification and disinfection	COD Calculated using IPCC factors for CH4, which for a AS is 7.5gCH4/KgCOD (inverse calculation based on the methane emissions calculated in the study). Assuming an ~85% COD reduction, which is an average reduction for AS. Carbon emissions calculated using Wang et al 2022, based on COD removed. Sludge production based on European averages.	Moussavi et al 2024
Activated sludge (AS10)	Standard AS process with anaerobic digestion	System expansion flows eliminated. Sludge handling without recovery calculated using the transportation of sludge to agricultural site, instead of assuming land application. Fossil carbon emissions calculated using Wang et al (2022) based on COD removed.	Foley et al (2010)
Activated sludge (AS11)	Standard AS process with nitrification stage and anaerobic digestion	System expansion flows eliminated. Sludge handling without recovery calculated using the transportation of sludge to agricultural site, instead of assuming land application. Fossil carbon emissions calculated using Wang et al (2022) based on COD removed.	Foley et al (2010)
Activated sludge (AS12)	AS process with extended aeration	Emissions to air calculated using IPCC factors. Since influent is not provided, this is estimated assuming a BOD:N:P ratio of 100:17:3. See SI-5 for calculations to obtain these ratios. Sludge based on average values.	Lopsik (2013)
Activated sludge (AS13)	Cyclic AS, with an anoxic-aerobic tank, secondary clarifier and disinfection step	Fossil carbon estimated using factors from Wang et al (2022). Construction from Doka (2003)	Pasciucco et al (2023)

Classified as Internal | Intern

Activated sludge (AS14)	Standard AS process, with UV radiation step and N/P precipitation step	Construction from Doka (2003)	Estevez et al. (2022)
Activated sludge (AS15)	Standard AS process with CWs as tertiary treatment	Fossil carbon and methane emissions calculated using IPCC factors, based on COD removed. Construction based on Doka (2003) and seludge generated based on average values.	Kalbar et al (2013)
Activated sludge (AS16)	Standard AS process, with two decanters and a disinfection unit	Methane calculated using IPCC factors, with a methane correction of 0,014. Construction from Doka (2003)	Ortiz et al (2007)
Anaerobic membrane bioreactor (AnMBR1)	AnMBR with trickling filter	Construction from Doka (2003)	Lijo et al (2017)
Anaerobic membrane bioreactor (AnMBR2)	AnMBR with 4-stage bardenpho as tertiary treatment. N limited at 10 mg/L	System expansion flows eliminated. Sludge handling without recovery calculated using the transportation of sludge to agricultural site, instead of assuming land application. Fossil carbon emissions calculated using Wang et al (2022) based on COD removed.	Foley et al (2010)
Anaerobic membrane bioreactor (AnMBR3)	AnMBR with 4-stage bardenpho as tertiary treatment. N limited at 10 mg/L and P limited at 5 mg/L	System expansion flows eliminated. Sludge handling without recovery calculated using the transportation of sludge to agricultural site, instead of assuming land application. Fossil carbon emissions calculated using Wang et al (2022) based on COD removed.	Foley et al (2010)
Anaerobic membrane bioreactor (AnMBR4)	AnMBR with 4-stage bardenpho as tertiary treatment. N limited at 10 mg/L and P limited at 1 mg/L	System expansion flows eliminated. Sludge handling without recovery calculated using the transportation of sludge to agricultural site, instead of assuming land application. Fossil carbon emissions calculated using	Foley et al (2010)

Classified as Internal | Intern

		Wang et al (2022) based on COD removed.	
Anaerobic membrane bioreactor (AnMBR5)	AnMBR with 4-stage bardenpho as tertiary treatment. N limited at 5 mg/L	System expansion flows eliminated. Sludge handling without recovery calculated using the transportation of sludge to agricultural site, instead of assuming land application. Fossil carbon emissions calculated using Wang et al (2022) based on COD removed.	Foley et al (2010)
Anaerobic membrane bioreactor (AnMBR6)	AnMBR with 4-stage bardenpho as tertiary treatment. N limited at 5 mg/L and P limited at 5 mg/L	System expansion flows eliminated. Sludge handling without recovery calculated using the transportation of sludge to agricultural site, instead of assuming land application. Fossil carbon emissions calculated using Wang et al (2022) based on COD removed.	Foley et al (2010)
Anaerobic membrane bioreactor (AnMBR7)	AnMBR with 4-stage bardenpho as tertiary treatment. N limited at 5 mg/L and P limited at 1 mg/L	System expansion flows eliminated. Sludge handling without recovery calculated using the transportation of sludge to agricultural site, instead of assuming land application. Fossil carbon emissions calculated using Wang et al (2022) based on COD removed.	Foley et al (2010)
Anaerobic membrane bioreactor (AnMBR8)	AnMBR with 4-stage bardenpho as tertiary treatment. N limited at 3 mg/L	System expansion flows eliminated. Sludge handling without recovery calculated using the transportation of sludge to agricultural site, instead of assuming land application. Fossil carbon emissions calculated using Wang et al (2022) based on COD removed.	Foley et al (2010)
Anaerobic membrane bioreactor (AnMBR9)	AnMBR with 4-stage bardenpho as tertiary treatment. N limited at 3 mg/L and P limited at 5 mg/L	System expansion flows eliminated. Sludge handling without recovery calculated using the transportation of sludge to agricultural site,	Foley et al (2010)

Classified as Internal | Intern

		instead of assuming land application. Fossil carbon emissions calculated using Wang et al (2022) based on COD removed.	
Anaerobic membrane bioreactor (AnMBR10)	AnMBR with 4-stage bardenpho as tertiary treatment. N limited at 3 mg/L and P limited at 1 mg/L	System expansion flows eliminated. Sludge handling without recovery calculated using the transportation of sludge to agricultural site, instead of assuming land application. Fossil carbon emissions calculated using Wang et al (2022) based on COD removed.	Foley et al (2010)
Anaerobic membrane bioreactor (AnMBR11)	BW/GW separations, with BW going to AnMBR with UV disinfection, and GW to a MBR	Original inventory assumes effluent is reused for irrigation. For the harmonisation, this assumption is eliminated and emissions to water are recalculated. Construction from Doka (2003)	Estevez et al (2022)
Bardenpho (BDP1)	5-stage BDP with extended aeration, N limited to 10 mg/L	System expansion flows eliminated. Sludge handling without recovery calculated using the transportation of sludge to agricultural site, instead of assuming land application.	Foley et al (2010)
Bardenpho (BDP2)	5-stage BDP with extended aeration, N limited to 10 mg/L and P to 5 mg/L	System expansion flows eliminated. Sludge handling without recovery calculated using the transportation of sludge to agricultural site, instead of assuming land application. Fossil carbon emissions calculated using Wang et al (2022) based on COD removed.	Foley et al (2010)
Bardenpho (BDP3)	5-stage BDP with extended aeration, N limited to 10 mg/L and P to 1 mg/L	System expansion flows eliminated. Sludge handling without recovery calculated using the transportation of sludge to agricultural site, instead of assuming land application. Fossil carbon	Foley et al (2010)

Classified as Internal | Intern

		emissions calculated using Wang et al (2022) based on COD removed.	
Bardenpho (BDP4)	5-stage BDP with extended aeration, N limited to 5 mg/L	System expansion flows eliminated. Sludge handling without recovery calculated using the transportation of sludge to agricultural site, instead of assuming land application. Fossil carbon emissions calculated using Wang et al (2022) based on COD removed.	Foley et al (2010)
Bardenpho (BDP5)	5-stage BDP with extended aeration, N limited to 5 mg/L and P to 5 mg/L	System expansion flows eliminated. Sludge handling without recovery calculated using the transportation of sludge to agricultural site, instead of assuming land application. Fossil carbon emissions calculated using Wang et al (2022) based on COD removed.	Foley et al (2010)
Bardenpho (BDP6)	5-stage BDP with extended aeration, N limited to 3 mg/L	System expansion flows eliminated. Sludge handling without recovery calculated using the transportation of sludge to agricultural site, instead of assuming land application. Fossil carbon emissions calculated using Wang et al (2022) based on COD removed.	Foley et al (2010)
Bardenpho (BDP7)	5-stage BDP with extended aeration, N limited to 3 mg/L and P to 5 mg/L	System expansion flows eliminated. Sludge handling without recovery calculated using the transportation of sludge to agricultural site, instead of assuming land application. Fossil carbon emissions calculated using Wang et al (2022) based on COD removed.	Foley et al (2010)
Bardenpho (BDP8)	5-stage BDP with extended aeration, N limited to 3 mg/L and P to 1 mg/L	System expansion flows eliminated. Sludge handling without recovery calculated using the transportation of	Foley et al (2010)

		sludge to agricultural site, instead of assuming land application. Fossil carbon emissions calculated using Wang et al (2022) based on COD removed.	
Bardenpho (BDP9)	5-stage BDP with extended aeration, N limited to 3 mg/L and P to 1 mg/L	System expansion flows eliminated. Sludge handling without recovery calculated using the transportation of sludge to agricultural site, instead of assuming land application. Fossil carbon emissions calculated using Wang et al (2022) based on COD removed.	Foley et al (2010)
Constructed Wetlands (CW1)	Standard CW	Emissions to water calculated assuming a BOD:N:P ratio of 100:17:3 (see SI-5 for more information) and average removal efficiency of CWs. Emissions to air calculated using IPCC guidelines and Wang et al (2022) factors for fossil carbon. Sludge based on average values for Europe.	Corbella et al (2017)
Constructed Wetlands (CW2)	CW with a gravel microbial fuel cell	Emissions to water calculated assuming a BOD:N:P ratio of 100:17:3 (see SI-5 for more information) and average removal efficiency of CWs. Emissions to air calculated using IPCC guidelines and Wang et al (2022) factors for fossil carbon. Sludge based on average values for Europe.	Corbella et al (2017)
Constructed Wetlands (CW3)	CW with a graphite microbial fuel cell	Emissions to water calculated assuming a BOD:N:P ratio of 100:17:3 (see SI-5 for more information) and average removal efficiency of CWs. Emissions to air calculated using IPCC guidelines and Wang et al (2022) factors for fossil carbon. Sludge based on average values for Europe.	Corbella et al (2017)

Classified as Internal | Intern

Constructed Wetlands (CW4)	Vertical flow CW followed by horizontal flow CW, with disinfection unit	Emissions to water calculated assuming a BOD:N:P ratio of 100:17:3 (see SI-5 for more information) and average removal efficiency of CWs. Sludge based on average values for Europe.	Garfi et al (2017)
Constructed Wetlands (CW5)	Vertical flow CW followed by horizontal flow CW	Fossil carbon calculated using IPCC factor. Transport of sludge based on sludge produced.	Risch et al (2021)
Constructed Wetlands (CW6)	Two-stage CW	Fossil carbon calculated using IPCC factor. Transport of sludge based on sludge produced.	Risch et al (2011)
Constructed Wetlands (CW7)	Free-flow CW with secondary settle and sub-superficial vertical flow WC	Fossil carbon calculated using Wang et al (2022) factor with IPCC fossil fraction factor. Sludge based on average European sludge production.	Resende et al (2019)
Membrane bioreactor (MBR1)	MBR with 4-stage BDP as tertiary treatment, limiting N to 10 mg/L	System expansion flows eliminated. Sludge handling without recovery calculated using the transportation of sludge to agricultural site, instead of assuming land application. Fossil carbon emissions calculated using Wang et al (2022) based on COD removed.	Foley et al (2010)
Membrane bioreactor (MBR2)	MBR with 4-stage BDP as tertiary treatment, limiting N to 10 mg/L and P to 5 mg/L	System expansion flows eliminated. Sludge handling without recovery calculated using the transportation of sludge to agricultural site, instead of assuming land application. Fossil carbon emissions calculated using Wang et al (2022) based on COD removed.	Foley et al (2010)
Membrane bioreactor (MBR3)	MBR with 4-stage BDP as tertiary treatment, limiting N to 10 mg/L and P to 5 mg/L	System expansion flows eliminated. Sludge handling without recovery calculated using the transportation of sludge to agricultural site, instead of assuming land	Foley et al (2010)

Classified as Internal | Intern

		application. Fossil carbon emissions calculated using Wang et al (2022) based on COD removed.	
Membrane bioreactor (MBR4)	MBR with 4-stage BDP as tertiary treatment, limiting N to 10 mg/L and P to 1 mg/L	System expansion flows eliminated. Sludge handling without recovery calculated using the transportation of sludge to agricultural site, instead of assuming land application. Fossil carbon emissions calculated using Wang et al (2022) based on COD removed.	Foley et al (2010)
Membrane bioreactor (MBR5)	MBR with 4-stage BDP as tertiary treatment, limiting N to 5 mg/L	System expansion flows eliminated. Sludge handling without recovery calculated using the transportation of sludge to agricultural site, instead of assuming land application. Fossil carbon emissions calculated using Wang et al (2022) based on COD removed.	Foley et al (2010)
Membrane bioreactor (MBR6)	MBR with 4-stage BDP as tertiary treatment, limiting N to 5 mg/L and P to 5 mg/L	System expansion flows eliminated. Sludge handling without recovery calculated using the transportation of sludge to agricultural site, instead of assuming land application. Fossil carbon emissions calculated using Wang et al (2022) based on COD removed.	Foley et al (2010)
Membrane bioreactor (MBR7)	MBR with 4-stage BDP as tertiary treatment, limiting N to 10 mg/L	System expansion flows eliminated. Sludge handling without recovery calculated using the transportation of sludge to agricultural site, instead of assuming land application. Fossil carbon emissions calculated using Wang et al (2022) based on COD removed.	Foley et al (2010)
Membrane bioreactor (MBR8)	MBR with 4-stage BDP as tertiary treatment, limiting	System expansion flows eliminated. Sludge handling without recovery calculated	Foley et al (2010)

Classified as Internal | Intern

	N to 5 mg/L and P to 1 mg/L	using the transportation of sludge to agricultural site, instead of assuming land application. Fossil carbon emissions calculated using Wang et al (2022) based on COD removed.	
Membrane bioreactor (MBR9)	MBR with 4-stage BDP as tertiary treatment, limiting N to 3 mg/L	System expansion flows eliminated. Sludge handling without recovery calculated using the transportation of sludge to agricultural site, instead of assuming land application. Fossil carbon emissions calculated using Wang et al (2022) based on COD removed.	Foley et al (2010)
Membrane bioreactor (MBR10)	Hollow-fiber MBR with a standard AS system	GHG emissions calculated using IPCC factors. No factor for MBR, so used those for AS instead. Construction from Doka (2003). Sludge production based on European averages.	Hospido et al (2012)
Membrane bioreactor (MBR11)	Hollow-fiber MBR with an Anoxic-Aerobic tank	GHG emissions calculated using IPCC factors. No factors for MBR or A-Aer tanks, so used CAS instead. Construction from Doka (2003). Sludge production based on European averages.	Hospido et al (2012)
Membrane bioreactor (MBR12)	Hollow-fiber MBR with an A2O tank	GHG emissions calculated using IPCC factors. No MBR factors so used A2O. Construction from Doka (2003). Sludge production based on European averages.	Hospido et al (2012)
Membrane bioreactor (MBR13)	MBR, immersed	Methane emissions added using IPCC emission factor, with an average value for aerobic plants, since MBR is not a specific category within the guidelines. Construction using Doka (2003)	Ortiz et al (2007)
Membrane bioreactor (MBR14)	MBR, external	Methane emissions added using IPCC emission factor, with an average value for	Ortiz et al (2007)

		aerobic plants, since MBR is not a specific category within the guidelines. Construction using Doka (2003)	
Modified Ludzack-Ettinger (MLE1)	Standard MLE with anaerobic digestion prior to sludge incineration	CO2 emissions calculated using factors from Wang et al (2022) and then applying the carbon fraction from the IPCC guidelines. Construction from Doka (2003). Sludge production based on European averages.	Morelli et al (2018)
Modified Ludzack-Ettinger (MLE2)	MLE with nitrification/denitrification step and anaerobic digestion prior to sludge incineration, limiting N to 20 mg/L	System expansion flows eliminated. Sludge handling without recovery calculated using the transportation of sludge to agricultural site, instead of assuming land application. Fossil carbon emissions calculated using Wang et al (2022) based on COD removed.	Foley et al (2010)
Modified Ludzack-Ettinger (MLE4)	MLE with nitrification/denitrification step and anaerobic digestion prior to sludge incineration, limiting N to 20 mg/L and P to 5 mg/L	System expansion flows eliminated. Sludge handling without recovery calculated using the transportation of sludge to agricultural site, instead of assuming land application. Fossil carbon emissions calculated using Wang et al (2022) based on COD removed.	Foley et al (2010)
Modified Ludzack-Ettinger (MLE5)	MLE with nitrification/denitrification step and anaerobic digestion prior to sludge incineration, limiting N to 20 mg/L and P to 1 mg/L	System expansion flows eliminated. Sludge handling without recovery calculated using the transportation of sludge to agricultural site, instead of assuming land application. Fossil carbon emissions calculated using Wang et al (2022) based on COD removed.	Foley et al (2010)
Modified Ludzack-Ettinger (MLE6)	MLE with nitrification/denitrification step and anaerobic digestion prior to sludge	System expansion flows eliminated. Sludge handling without recovery calculated using the transportation of sludge to agricultural site,	Foley et al (2010)

	incineration, limiting N to 10 mg/L	instead of assuming land application. Fossil carbon emissions calculated using Wang et al (2022) based on COD removed.	
Modified Ludzack-Ettinger (MLE7)	MLE with nitrification/denitrification step and anaerobic digestion prior to sludge incineration, limiting N to 10 mg/L and P to 5 mg/L	System expansion flows eliminated. Sludge handling without recovery calculated using the transportation of sludge to agricultural site, instead of assuming land application. Fossil carbon emissions calculated using Wang et al (2022) based on COD removed.	Foley et al (2010)
Modified Ludzack-Ettinger (MLE8)	MLE with nitrification/denitrification step and anaerobic digestion prior to sludge incineration, limiting N to 10 mg/L and P to 1 mg/L	System expansion flows eliminated. Sludge handling without recovery calculated using the transportation of sludge to agricultural site, instead of assuming land application. Fossil carbon emissions calculated using Wang et al (2022) based on COD removed.	Foley et al (2010)
Modified Ludzack-Ettinger (MLE9)	MLE with nitrification/denitrification step and anaerobic digestion prior to sludge incineration, limiting N to 5 mg/L	System expansion flows eliminated. Sludge handling without recovery calculated using the transportation of sludge to agricultural site, instead of assuming land application. Fossil carbon emissions calculated using Wang et al (2022) based on COD removed.	Foley et al (2010)
Modified Ludzack-Ettinger (MLE10)	MLE with nitrification/denitrification step and anaerobic digestion prior to sludge incineration, limiting N to 5 mg/L and P to 5 mg/L	System expansion flows eliminated. Sludge handling without recovery calculated using the transportation of sludge to agricultural site, instead of assuming land application. Fossil carbon emissions calculated using Wang et al (2022) based on COD removed.	Foley et al (2010)
Modified Ludzack-	MLE with nitrification/denitrification	System expansion flows eliminated. Sludge handling	Foley et al (2010)

Classified as Internal | Intern

Ettinger (MLE11)	step and anaerobic digestion prior to sludge incineration, limiting N to 5 mg/L and P to 1 mg/L	without recovery calculated using the transportation of sludge to agricultural site, instead of assuming land application. Fossil carbon emissions calculated using Wang et al (2022) based on COD removed.	
Oxidation Ditch (OD1)	Standard OD	Flows for fertiliser recovery from land application eliminated. Construction from Doka (2003). Sludge production based on European averages.	Liu et al (2018)
Oxidation Ditch (OD2)	OD coupled with CW, fed with alum sludge, to enhance biosorption of pollutants.	Flows for fertiliser recovery from land application eliminated. Construction from Doka (2003). Sludge production based on European averages. Alum sludge, which is added at the operational stage, is not part of ecoinvent 3.9.1, but it's part of ecoinvent 3.12, thus the emissions associated with it were added manually as a biosphere flow. Since the system requires 4kg of alum sludge per m3 of wastewater, the emissions were multiplied by 4.	Liu et al (2018)
Oxidation Ditch (OD3)	Oxidation ditch, with return activatedsludge, and clarification and disinfection tertiary step	COD emissions calculated using a reverse factor for CH_4. CO_2 emissions based on COD removed, calculated using factors from Wang et al (2022), and fossil fraction estimated using IPCC factor.	Moussavi et al (2024)
Oxidation Ditch (OD4)	Oxidation ditch, with return activatedsludge, and clarification and disinfection tertiary step	COD emissions calculated using a reverse factor for CH_4. CO_2 emissions based on COD removed, calculated using factors from Wang et al (2022), and fossil fraction estimated using IPCC factor. Production of vitrified clay pipe based on Vahidi et al (2016)	Moussavi et al (2024)

Classified as Internal | Intern

Oxidation Ditch (OD5)	Oxidation ditch, with return activatedsludge, and clarification and disinfection tertiary step	COD emissions calculated using a reverse factor for CH_4. CO_2 emissions based on COD removed, calculated using factors from Wang et al (2022), and fossil fraction estimated using IPCC factor. Production of vitrified clay pipe based on Vahidi et al (2016)	Moussavi et al (2024)
Oxidation Ditch (OD6)	Oxidation ditch, with return activatedsludge, and clarification and disinfection tertiary step	COD emissions calculated using a reverse factor for CH_4. CO_2 emissions based on COD removed, calculated using factors from Wang et al (2022), and fossil fraction estimated using IPCC factor. Production of vitrified clay pipe based on Vahidi et al (2016)	Moussavi et al (2024)
Oxidation Ditch (OD7)	Oxidation ditch, with return activatedsludge, and clarification and disinfection tertiary step	COD emissions calculated using a reverse factor for CH_4. CO_2 emissions based on COD removed, calculated using factors from Wang et al (2022), and fossil fraction estimated using IPCC factor. Production of vitrified clay pipe based on Vahidi et al (2016)	Moussavi et al (2024)
Oxidation Ditch (OD8)	Oxidation ditch with extended aeration, with a nitrification/denitrification step, limiting N to 10 mg/L	System expansion flows eliminated. Sludge handling without recovery calculated using the transportation of sludge to agricultural site, instead of assuming land application. Fossil carbon emissions calculated using Wang et al (2022) based on COD removed.	Foley et al (2010)
Oxidation Ditch (OD9)	Oxidation ditch with extended aeration, with a nitrification/denitrification step, limiting N to 10 mg/L and P to 5 mg/L	System expansion flows eliminated. Sludge handling without recovery calculated using the transportation of sludge to agricultural site, instead of assuming land application. Fossil carbon emissions calculated using	Foley et al (2010)

Classified as Internal | Intern

		Wang et al (2022) based on COD removed.	
Oxidation Ditch (OD10)	Oxidation ditch with extended aeration, with a nitrification/denitrification step, limiting N to 10 mg/L and P to 1 mg/L	System expansion flows eliminated. Sludge handling without recovery calculated using the transportation of sludge to agricultural site, instead of assuming land application. Fossil carbon emissions calculated using Wang et al (2022) based on COD removed.	Foley et al (2010)
Oxidation Ditch (OD11)	Oxidation ditch with extended aeration, with a nitrification/denitrification step, limiting N to 5 mg/L	System expansion flows eliminated. Sludge handling without recovery calculated using the transportation of sludge to agricultural site, instead of assuming land application. Fossil carbon emissions calculated using Wang et al (2022) based on COD removed.	Foley et al (2010)
Oxidation Ditch (OD12)	Oxidation ditch with extended aeration, with a nitrification/denitrification step, limiting N to 5 mg/L and P to 5 mg/L	System expansion flows eliminated. Sludge handling without recovery calculated using the transportation of sludge to agricultural site, instead of assuming land application. Fossil carbon emissions calculated using Wang et al (2022) based on COD removed.	Foley et al (2010)
Oxidation Ditch (OD13)	Oxidation ditch with extended aeration, with a nitrification/denitrification step, limiting N to 5 mg/L and P to 1 mg/L	System expansion flows eliminated. Sludge handling without recovery calculated using the transportation of sludge to agricultural site, instead of assuming land application. Fossil carbon emissions calculated using Wang et al (2022) based on COD removed.	Foley et al (2010)
Oxidation Ditch (OD14)	Oxidation ditch with extended aeration, with a nitrification/denitrification step, limiting N to 3 mg/L	System expansion flows eliminated. Sludge handling without recovery calculated using the transportation of sludge to agricultural site,	Foley et al (2010)

		instead of assuming land application. Fossil carbon emissions calculated using Wang et al (2022) based on COD removed.	
Oxidation Ditch (OD15)	Oxidation ditch with extended aeration, with a nitrification/denitrification step, limiting N to 3 mg/L and P to 5 mg/L	System expansion flows eliminated. Sludge handling without recovery calculated using the transportation of sludge to agricultural site, instead of assuming land application. Fossil carbon emissions calculated using Wang et al (2022) based on COD removed.	Foley et al (2010)
Oxidation Ditch (OD16)	Oxidation ditch with extended aeration, with a nitrification/denitrification step, limiting N to 3 mg/L and P to 1 mg/L	System expansion flows eliminated. Sludge handling without recovery calculated using the transportation of sludge to agricultural site, instead of assuming land application. Fossil carbon emissions calculated using Wang et al (2022) based on COD removed.	Foley et al (2010)
Sequence batch reactor (SBR1)	Gray and blackwater separation, fed into a SBR and a UASB	Flows are adjusted to the FU of 1cubic meter, transformed from PE/yr in the original paper. This is done using 200 L/PE/d as a conversion factor as part of the harmonisation process. The study assumed 145 L/PE.d. Emissions and processes form fertiliser application excluded. Food waste has also been excluded, since it doesn't play a role in the water treatment, and thus biogas recovery reduced based only on wastewater-related values. Instead of land application, sludge is assumed to be incinerated. Pasteurisation step eliminated. GHG emissions calculated using IPCC and Wang et al (2022) for fossil carbon dioxide.	Lima et al (2023)

Sequence batch reactor (SBR2)	SBR followed by clarification and disinfection	COD emissions calculated using a reverse factor for CH_4. CO_2 emissions based on COD removed, calculated using factors from Wang et al (2022), and fossil fraction estimated using IPCC factor. Production of vitrified clay pipe based on Vahidi et al (2016)	Moussavi et al (2024)
Sequence batch reactor (SBR3)	SBR followed by clairfication and disinfection	COD emissions calculated using a reverse factor for CH_4. CO_2 emissions based on COD removed, calculated using factors from Wang et al (2022), and fossil fraction estimated using IPCC factor.	Moussavi et al (2024)
Sequence batch reactor (SBR4)	SBR followed by clairfication and disinfection	COD emissions calculated using a reverse factor for CH_4. CO_2 emissions based on COD removed, calculated using factors from Wang et al (2022), and fossil fraction estimated using IPCC factor.	Moussavi et al (2024)
Sequence batch reactor (SBR5)	SBR followed by clairfication and disinfection	COD emissions calculated using a reverse factor for CH_4. CO_2 emissions based on COD removed, calculated using factors from Wang et al (2022), and fossil fraction estimated using IPCC factor.	Moussavi et al (2024)
Sequence batch reactor (SBR6)	SBR followed by clairfication and disinfection	COD emissions calculated using a reverse factor for CH_4. CO_2 emissions based on COD removed, calculated using factors from Wang et al (2022), and fossil fraction estimated using IPCC factor. Production of vitrified clay pipe based on Vahidi et al (2016)	Moussavi et al (2024)
Sequence batch reactor (SBR7)	SBR followed by clairfication and disinfection	COD emissions calculated using a reverse factor for CH_4. CO_2 emissions based on COD removed, calculated using factors from Wang et al (2022), and fossil fraction	Moussavi et al (2024)

		estimated using IPCC factor. Production of vitrified clay pipe based on Vahidi et al (2016)	
Sequence batch reactor (SBR8)	Standard SBR	Values for Methane a nitrous oxides are based on literature values, so these were calculated based on primary influent/effluent data. CO_2 is calculated using Wang et al (2022) factor and then applying IPCC ratio for fossil carbon.	Kohlheb et al (2020)
Upflow anaerobic sludge blanket (UASB1)	UASB, with a trickling filter and biogas generation	CO_2 emissions based on COD removed, calculated using factors from Wang et al (2022), and fossil fraction estimated using IPCC factor. Sludge assumed to be incinerated instead of landfilled. Transport from construction based on Doka (2003)	Vasalle et al (2023)
Upflow anaerobic sludge blanket (UASB2)	UASB, with a polishing pond and biogas generation	CO_2 emissions based on COD removed, calculated using factors from Wang et al (2022), and fossil fraction estimated using IPCC factor. Sludge assumed to be incinerated instead of landfilled. Transport from construction based on Doka (2003)	Vasalle et al (2023)
Upflow anaerobic sludge blanket (UASB3)	UASB as main treatment, followed by a SBR, with a biotrickling filter for biogas production and added food waste as carbon source	All carbon is assumed to be biogenic – this is changed using factors from the updated IPCC guidelines (2019). Construction from Doka (2003)	Lijo et al (2017)
Upflow anaerobic sludge blanket (UASB4)	UASB followed by a CW	CO_2 emissions based on COD removed, calculated using factors from Wang et al (2022), and fossil fraction estimated using IPCC factor. Sludge assumed to be incinerated instead of landfilled. Transport from	Vasalle et al (2023)

Classified as Internal | Intern

		construction based on Doka (2003)	
Upflow anaerobic sludge blanket (UASB5)	UASB followed by a CW	GHG emissions calculated using IPCC emission factors. Transport of sludge added based on sludge produced.	Lopes et al (2020)

S9. LCA Results

In this section we present the LCA results for all impact categories, according to the impact assessment ReCiPe Midpoint 2016. In the main study we focused on the most important categories for wastewater according to recent reviews: climate change and eutrophication (Corominas et al., 2020). Ecotoxicity was not included in the main study due to lack of data on heavy metal and pathogen transfer in water discharge and sludge application. A more in-depth view of the results can be see in the excel file in the SI.

Classified as Internal | Intern

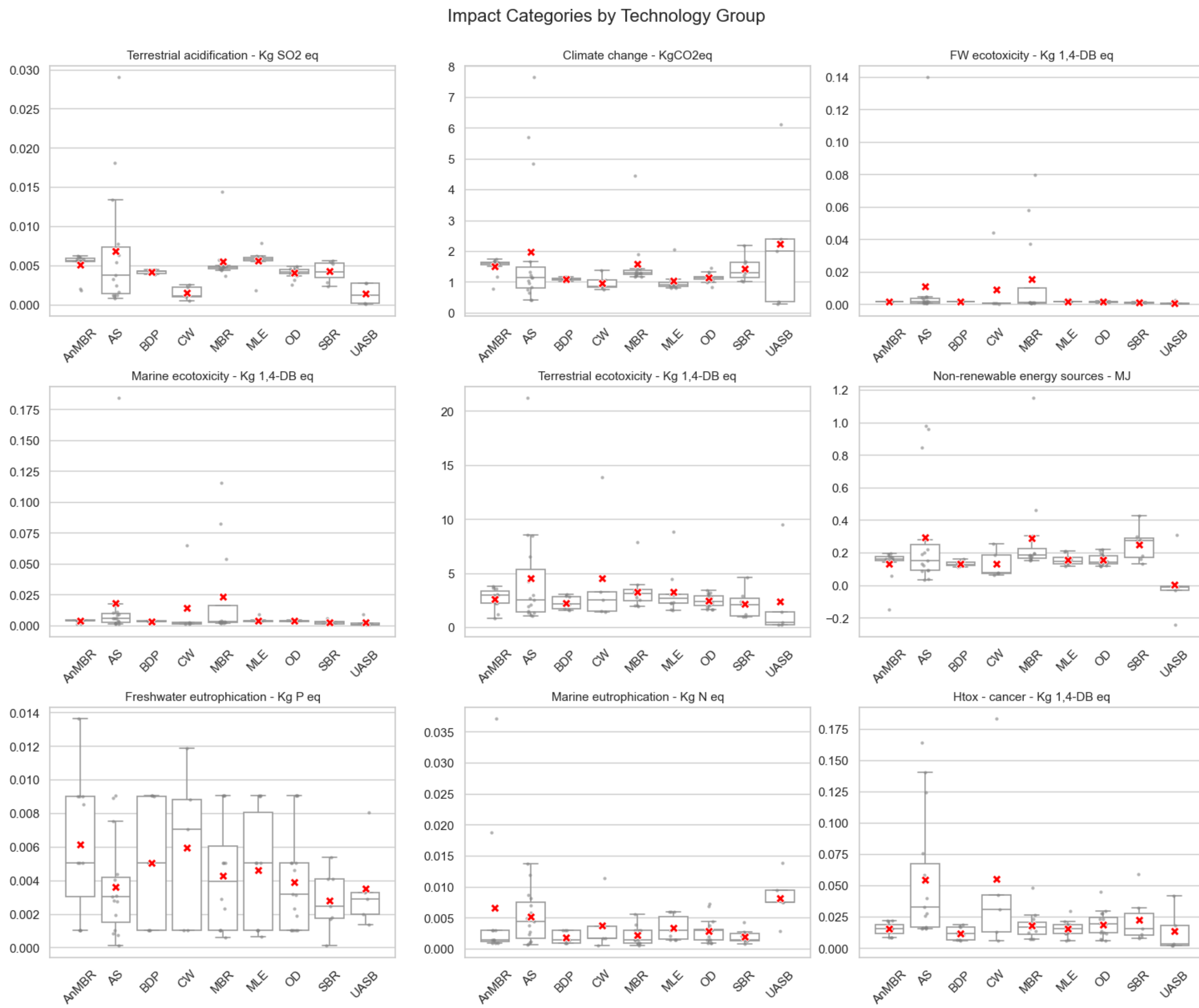

Classified as Internal | Intern

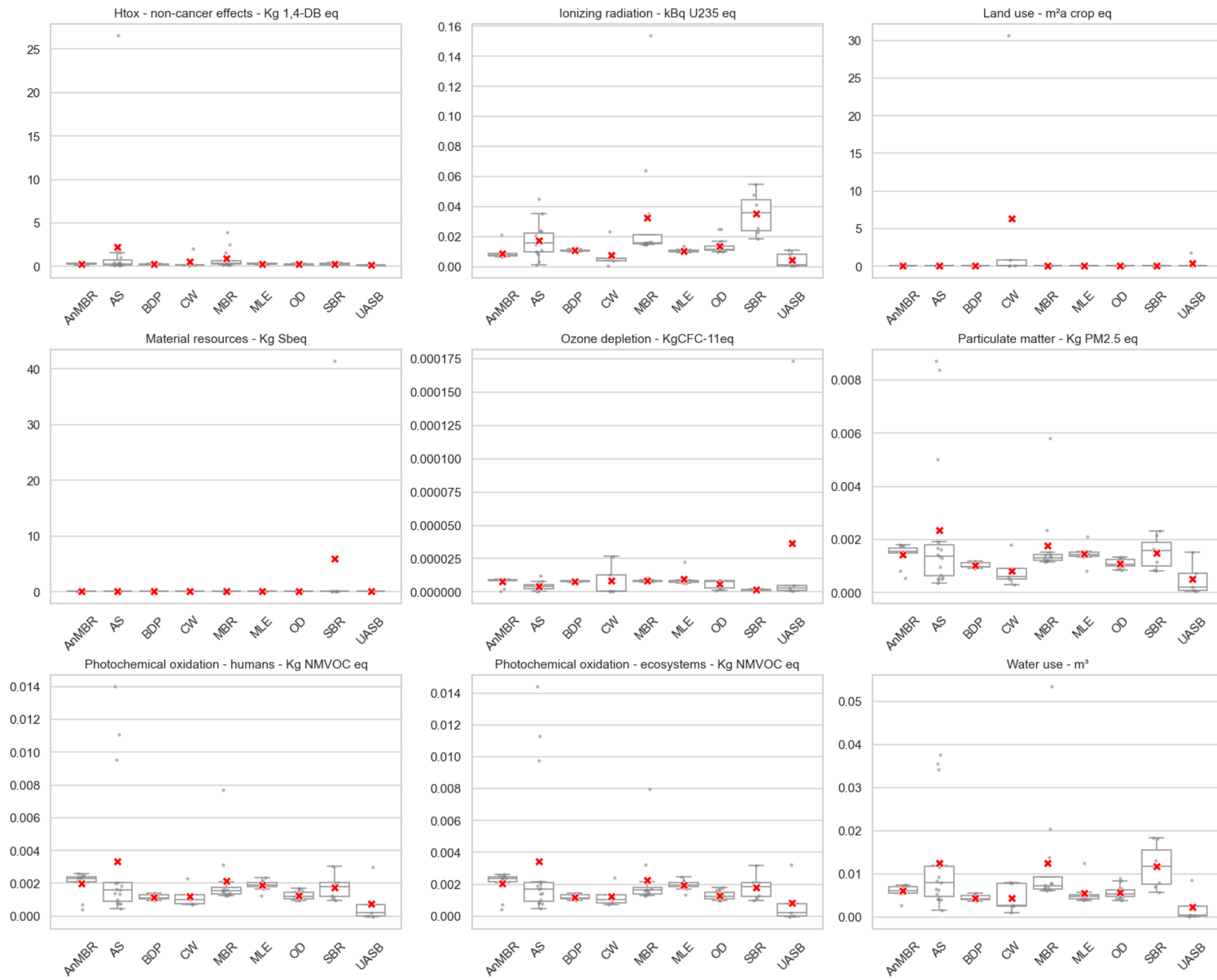

Figure S1. LCA results for 16 impact categories, according to ReCiPe Midpoint 2016

S10. Sensitivity analysis using economic allocation to handle multifunctionality

We carried out a sensitivity analysis for handling of multifunctional processes using economic allocation instead of substitution which is the default option for modeling in our work. To perform the economic allocation, we followed Guinée et al., (2004). We first estimate the price for wastewater treatment and for the other functional flows within each unit process. We then calculate an allocation factor based on the revenues of each functional flow determined by the price and amount. Once this is done, the share of the impacts of each multifunctional unit process are allocated using the calculated factors, with the corresponding share of impacts being allocated to the wastewater and to the other functional flow(s) (for example, NPK fertilizer). For more detail, see the examples below and the python scripts used in this calculation, available in the repository.

First, we selected prices available in market data and scientific literature. For the price of treating wastewater, we used existing life cycle costing analysis, although these values are often given in ranges and thus an average had to be estimated. Since there is no real consensus and prices will be determined based on system and location, an approximation must be based in values found in the literature. For example, in Jiménez-Benítez et al., (2023) it is discussed that costs per m3 in MBRs range from -0.2 € to -0.45€. Pryce et al., (2022) estimated an average price of -0.31 $/m^3 (~ -0.27 €/m^3) for small-scale wastewater treatment, and Çankaya & Pekey, (2024) calculated a range between -0.21 €/m^3 and - 0.53 €/m^3 for biological and enhanced biological wastewater treatment. In another recent study published by Moral Pajares et al., (2019), it was estimated that the average price per m^3 for WWT facilities ranged from -0.19€ to -0.50€, with an estimated average of -0.37 €/m^3. Based on these results, a rounded value of -0.3 €/m^3 was used for the price of treating wastewater. This value is used to estimate the price of the waste flows produced during the WWT. For example, there is no value for struvite found in WW, but this can be precipitated and further processes to obtain NPK fertilizer. In these cases, the price of struvite was estimated based on the price of treating water; for example, in a case in which a system recovers 0.5 kg of struvite per m^3 of treated water, we assumed a cost of 0.15 €/kg$_{struvite}$ (0.3 €/m^3 • 0.5 kg$_{struvite}$ = 0.15 €/kg$_{struvite}$). This approach provides a reasonable proxy cost for a resource with no real market data.

Sludge handling prices were obtained from Domini et al., (2022), who estimated the variation in costs for sludge treatment in Italy. Although the costs presented in this study are not representative for all of Europe, they are the most recent estimation available for such costs. The authors report an average 212 €/t of sludge for incineration, which is 0.21 €/kg of sludge.

Broadly, the resources recovered from WWT are fertilizer, energy and water. As mentioned, during the wastewater treatment and before the sludge handling, struvite can be precipitated to recover NPK fertilizer. In these cases, market values for N and P-based fertilizer were used to estimate revenue, available from the European Commission's Agridata (European Comission, 2025).

Regarding energy, the standard way to recover it is through anaerobic digestion of the sludge, although incineration of sludge after full dewatering can also recover energy in the form of heat and electricity. Heat pumps can also be installed at the discharge level to recover energy from the effluent's temperature. In all these cases, we assumed a 1:1 replacement ratio and used average market price for electricity in western Europe. According to Eurostat, the average price of electricity was around 0.27 €/kWh (Eurostat, 2025). For heat, we used the findings from Fallahnejad et al., (2024), who estimate an

average cost of 32 €/MWh, which is equivalent to 0.009 €/MJ. A final resource recovered is biodiesel. For this, we used a unitary price reported by Khan et al (2021), where the revenue of biodiesel recovered from WWT is 1.59 €/kg. To estimate the cost of biogas we used the value reported by Gupta & Khatiwada, (2024), which is 0.08 €/m^3.

To calculate the revenue (Rev) of each functional flow i, we used the following formula:

$$Rev_{FF_i} = P \cdot Q_i$$

Equation 9

Where the revenue of a functional flow i (Rev_{FF_i}) is the unit price of the functional flow i (P_i), multiplied by the quantity of the functional flow i (Q_i).

To calculate the allocation factor (AF) for functional flow i, we used the following formula:

$$AF_i = \frac{Rev_{FF_i}}{\sum_{i=1}^{n}(Rev_{FF_i})}$$

Equation 10

The calculations and LCAs were performed using Brightway 2.0, and the scripts are available in the GitHub repository (https://github.com/Milo-Solis/globla_harmonisation_mWWM.git), also accessible as a separate SI file.

We tested for sensitivity regarding handling of multifunctionality in two cases where we observed the highest level of resource recovery. First, we applied economic allocation to AnMBR1, which has organic waste as external carbon source coupled with a biotrickling filter from which biogas is recovered for household heating. Sludge is incinerated, which recovers some energy in the form of heat and electricity. The allocation factors are presented in **Table S2** and the system boundary in **Figure S2**. The blue shades display the multifunctional unit processes, where “G” stands for “good” and “W” for “waste”.

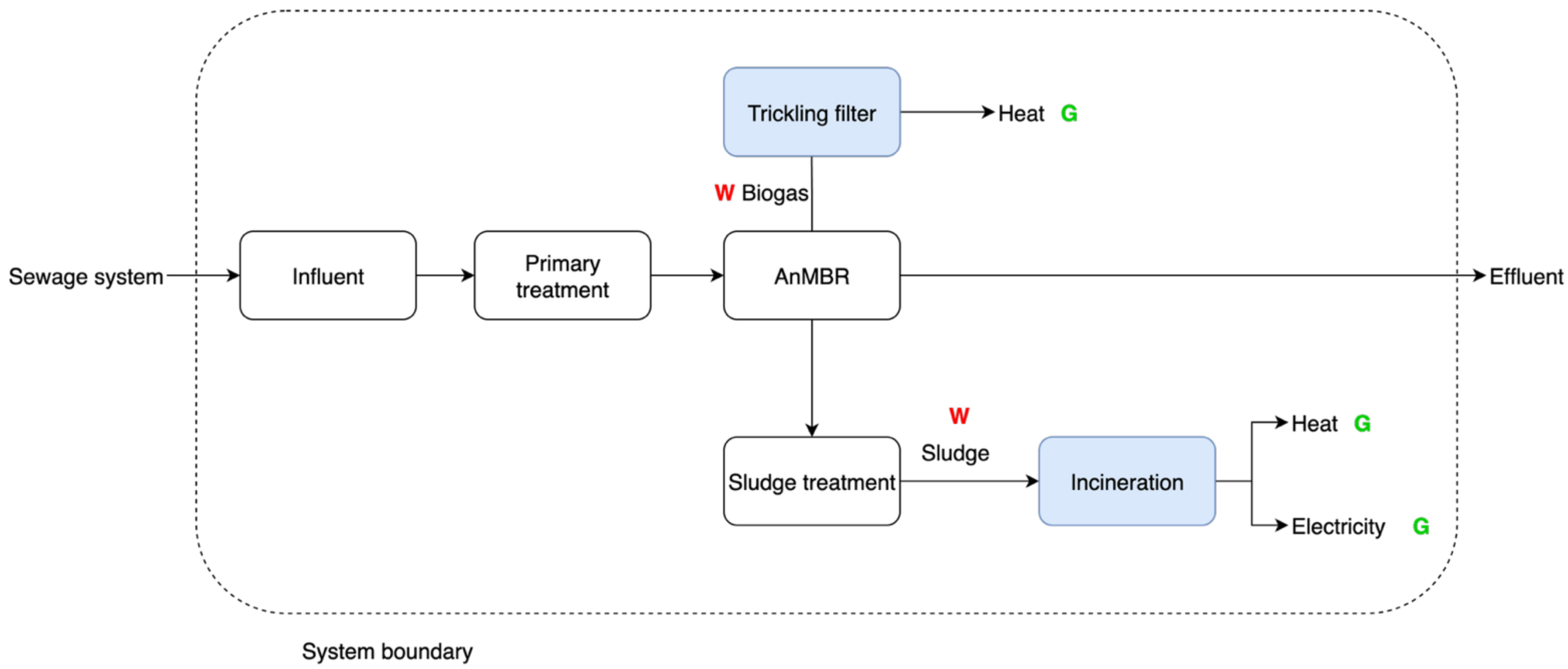

Figure S2. System boundary of AnMBR1 using economic allocation

Table S2. Values used for economic allocation of AnMBR1

Unit process and functional flows	Quantity (Q_i)	Unit cost (C_i)	Total revenue/costs (Rev_{FF_i})	Allocation factor (AF_i)
**Biogas processing with heat recovery**				
**Biogas**	-0.24 m³	-0.08 €/m³	0.019 €	0.21
**Heat**	7.81 MJ	0.009 €/MJ	0.07 €	0.79
**total**			0.089 €	1
**Sludge incineration**				
**Sludge**	-0.789 kg	-0.21 €/kg	0.16 €	0.93
**Electricity**	0.03 kWh	0.27 €/kWh	0.0081 €	0.05
**Heat**	0.4 MJ	0.009 €/MJ	0.0036 €	0.02
**total**			0.17 €	1

Using the logic in the above formulas, we isolated the multifunctional unit processes and carried out an LCA using the AF presented in **Table S2**. The results comparing economic allocation and substitution can be seen below in **Figure S3** and in **Table S3**.

Classified as Internal | Intern

Table S3. Climate change results of AnMBR1, using substitution and economic allocation

Unit process	Substitution	Economic allocation
**AnMBR WWT***	1.17 kg CO_2-eq	1.17 kg CO_2-eq
**Biogas processing with heat recovery**	-0.044 kg CO_2-eq	0.040 kg CO_2-eq
**Sludge disposal, incineration**	0.087 kg CO_2-eq	0.081 kg CO_2-eq
**total**	1.21 kg CO_2-eq	1.29 kg CO_2-eq

* AnMBR WWT refers to the treatment process, which is the same in both cases since there is no multifunctionality, but is calculated separate from the other unit processes.

Overall, using economic allocation leads to a slight increase in climate change impacts (**Table S3 and Figure S3**). When applying substitution, all the benefits of the heat recovery are allocated to the WWTP and are outweighing the environmental impacts of the recovery process, which leads to a reduction in climate change impacts of the overall treatment process. The electricity and energy recovered from sludge incineration is not sufficient to offset the environmental impacts of this process. In this case, the difference between the two approaches is minimal and does not meaningfully affect the overall conclusion.

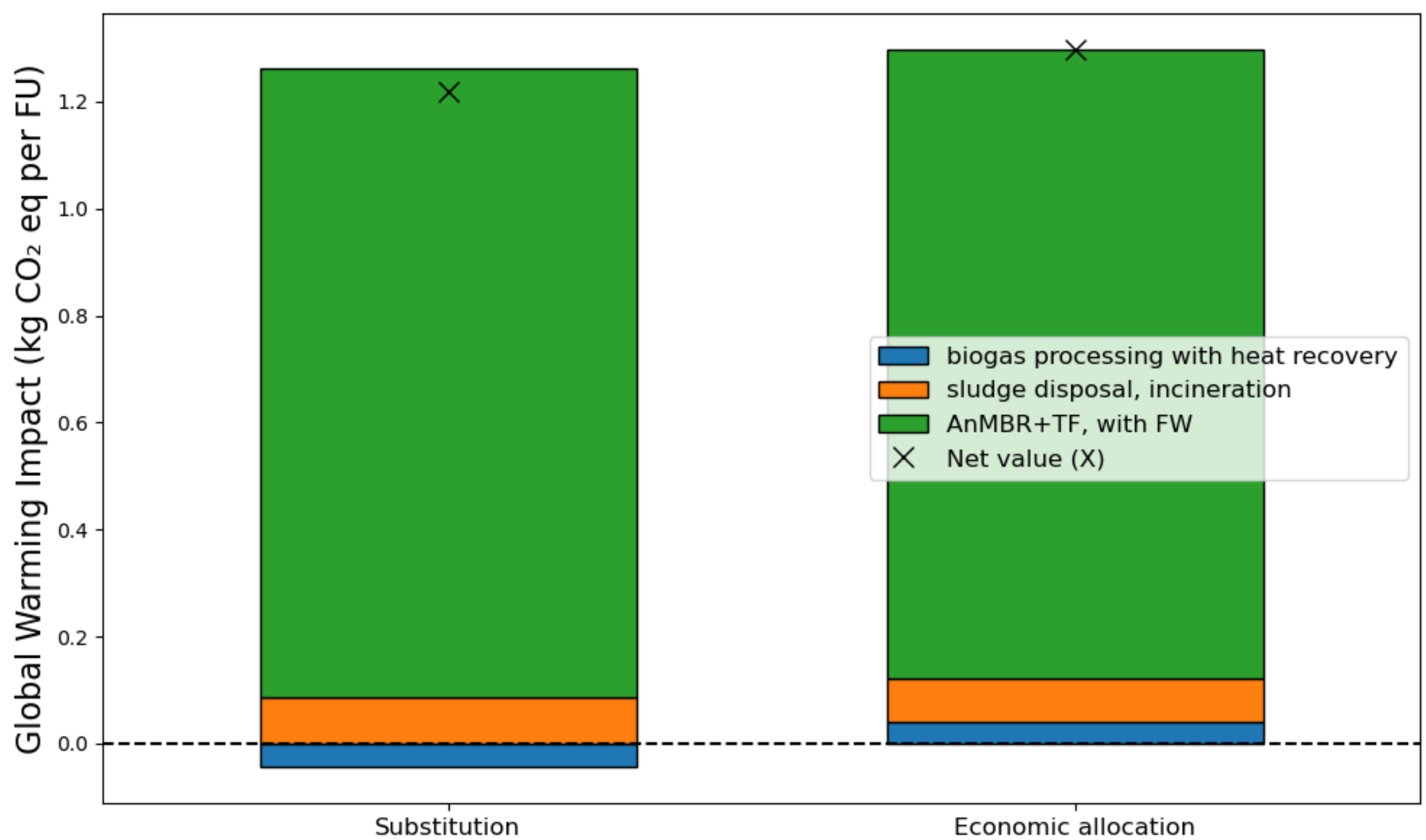

Figure S3. Climate change results for AnMBR1 using substitution and economic allocation. The “X” displays the net value under substitution.

Classified as Internal | Intern

We also carried out the same sensitivity analysis for SBR1 (SBR+UASB, GW/BW separation). The system boundary and flow diagram is presented in **Figure S4**. The allocation factors are presented in **Table S4**. The results of the sensitivity analysis are presented in **Table S5** and **Figure S5**.

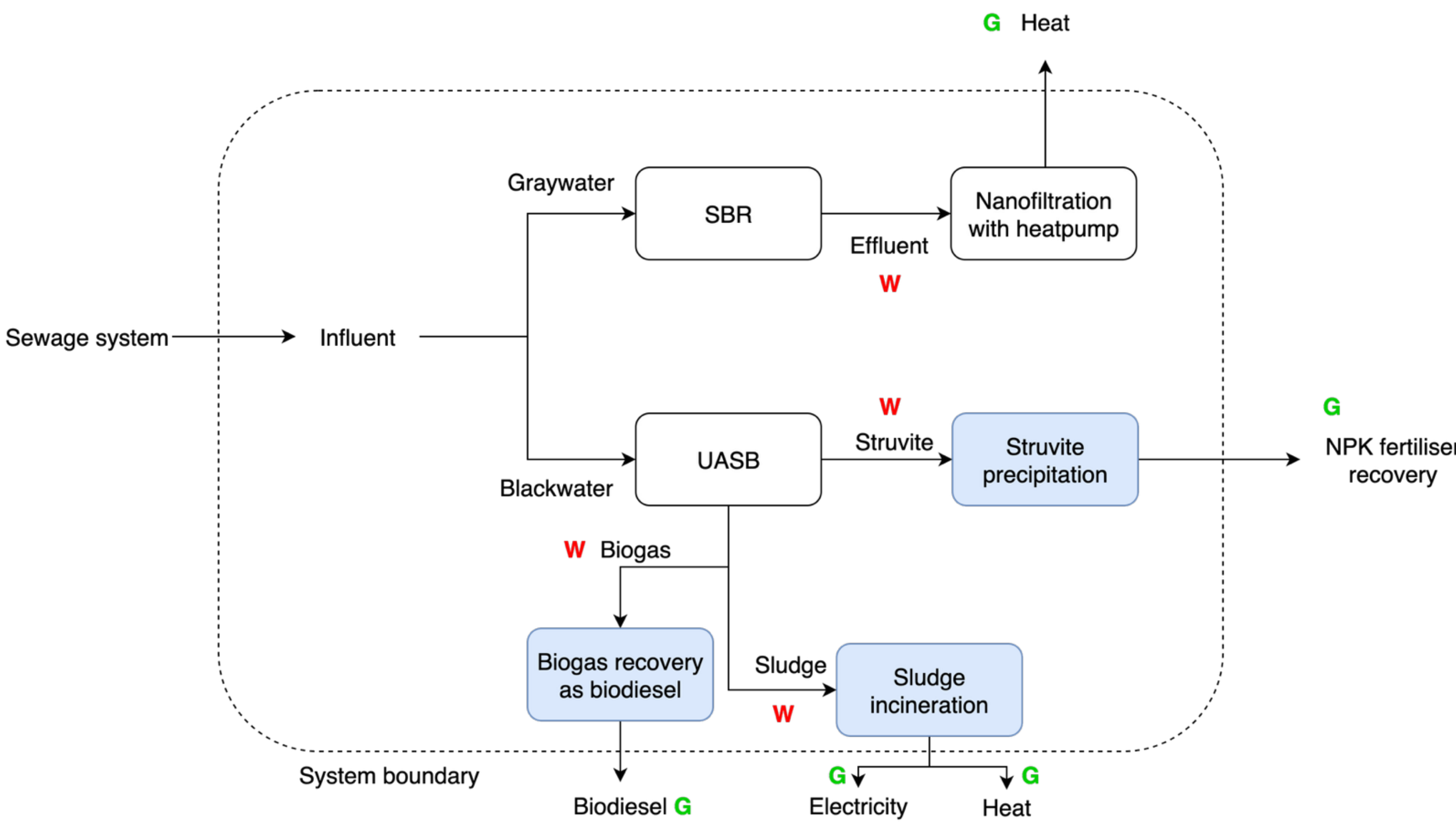

Figure S4. System boundary for the sensitivity analysis of SBR1 using economic allocation.

Table S4. Values for economic allocation factors for SBR1

Unit process and functional flows	Quantity (Q_i)	Unit cost (C_i)	Total revenue/costs (Rev_{FF_i})	Allocation factor (AF_i)
**Nanofiltration with heat pump**				
**Greywater***	-1 m^3	-0.3 €/m^{3*}	0.3 €	0.62
**Heat**	20.7 MJ	0.009 €/MJ	0.18€	0.38
total			*0.48 €*	*1*
**Struvite precipitation**				
**Struvite**	-0.009 Kg/m^3	-0.3 €/kg	0.0027 €	0.1
**NPK Fertiliser**	0.054 kg		0.023 €	0.9

**Nitrogen**	0.04 kg	0.39 €/kg	0.015 €	
**Phosphorus**	0.014 kg	0.62 €/kg	0.008 €	
total			*0.025 €*	*1*
**Biogas recovery as biodiesel**				
**Biogas**	-0.15 m^3	-0.08 €/m^3	0.012 €	0.09
**Biodiesel**	0.075 kg	1.59 €/kg	0.119 €	0.91
total			*0.131*	*1*
**Sludge disposal, incineration**				
**Sludge**	-0.58 kg	-0.21 €/kg	0.12 €	0.93
**Electricity**	0.024 kWh	0.27 €/kWh	0.0064 €	0.05
**Heat**	0.29 MJ	0.009 €/MJ	0.0026 €	0.02
total			*0.12 €*	*1*

* Price for this flow calculated as the price of treating and cleaning wastewater (see above for a more in-depth explanation)

In this case, economic allocation result in lower impacts (1.02 kg CO_2-eq) allocated to the WWTP when compared with substitution (1.28 kg CO_2-eq). Even though using substitution means that some emissions are mitigated with the heat and biogas recovery, in economic allocation around 90% of the impacts associated with NPK recovery are not part of the WWTP. This stage is a considerable contributor to climate change impacts under substitution, and although recovering NPK fertilizer may be advantageous from a resource depletion perspective, it results in higher climate change impacts.

In this case, handling multifunctionality using economic allocation results in 0.24 kg CO_2-eq allocated less to the WWT.

Table S5. Results for SBR1, using substitution and economic allocation to handle multifunctionality

Unit process	Substitution	Economic allocation
**SBR+UASB WWT***	0.87 kg CO_2-eq	0.87 kg CO_2-eq
**Biogas recovery as biodiesel**	-0.05 kg CO_2-eq	0.002 kg CO_2-eq
**Sludge disposal, incineration**	0.062 kg CO_2-eq	0.06 kg CO_2-eq
**NPK recovery**	0.99 kg CO_2-eq	0.10 kg CO_2-eq
**Nanofiltration with heatpump**	-0.59 kg CO_2-eq	0.021kg CO_2-eq
**total**	1.28 kg CO_2-eq	1.05 kg CO_2-eq

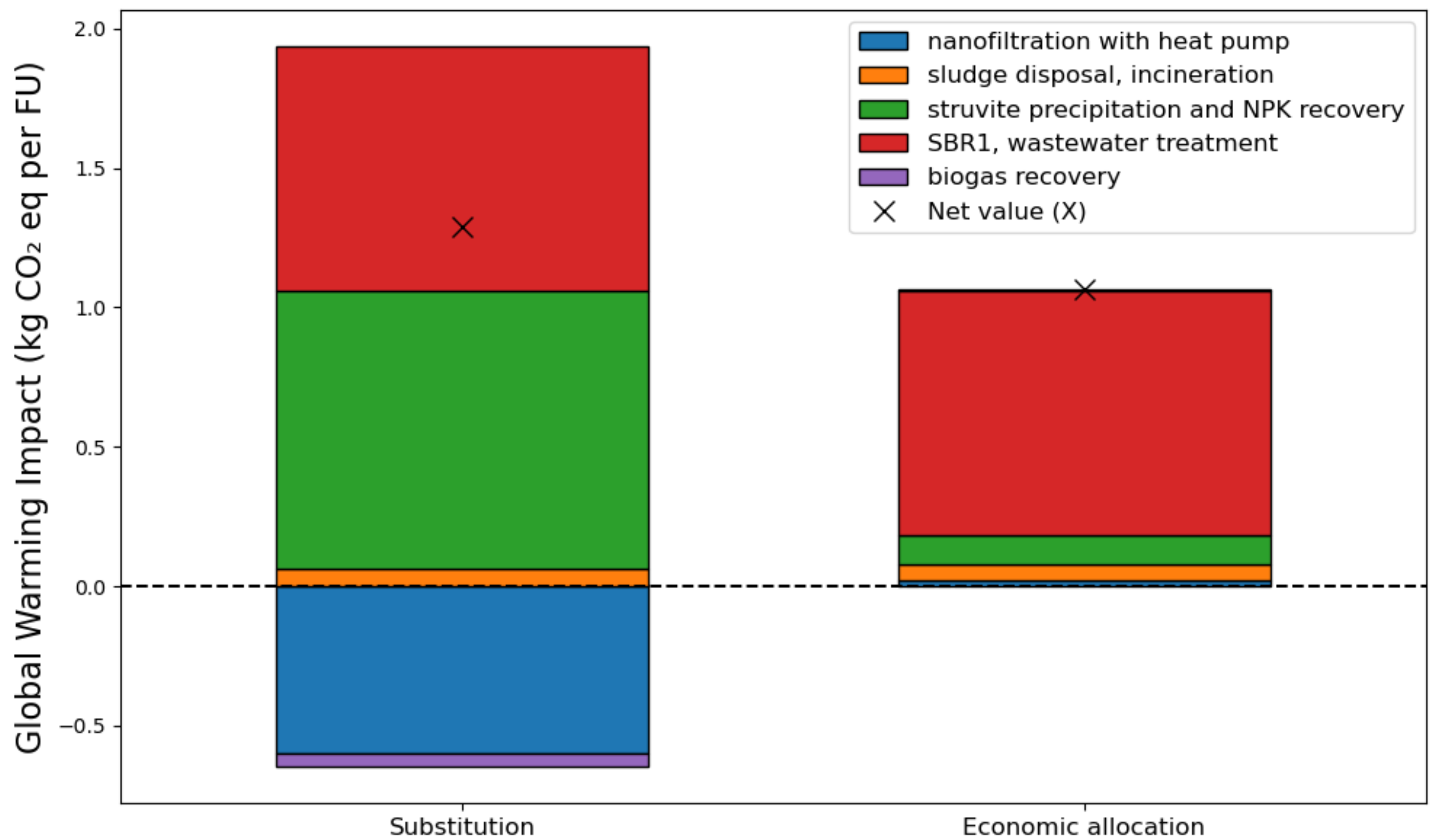

Figure S5. Climate change impacts of SBR1 using substitution and economic allocation. The "X" displays the net value under substitution.

In both cases, substitution yields either a more conservative estimate or one that is comparable to economic allocation. Given that the study aims to provide an overarching assessment of numerous mWWM systems, we consider a conservative approach less likely to be misleading or overly optimistic. This reasoning supports our decision to address multifunctionality using substitution in this study. However, we acknowledge that there are cases where handling multifunctional processes with substitution could lead to lower results compared to handling via allocation.

S11. Electricity mix sensitivity analysis

Since the geographical location of the harmonization was set in Europe, an average European electricity mix was used as the background process. Given the high renewable energy penetration in the European grid, this modelling choice can have disproportionate influence on the overall climate change impacts. To test this, a sensitivity analysis was carried out, replacing average European electricity with average Chinese one. Despite also having substantial renewable energy penetration in the grid, sectors of Chinese energy generation are still reliant on fossil sources.

To test the impact of this background process, two technology groups were chose: SBRs and AnMBRs. The former because it displays high climate change impacts associated with

Classified as Internal | Intern

energy use; the latter, because its climate change impacts are predominantly dominated by direct GHG emissions, chemical dosing and sludge handling, with lower emissions being linked to electricity.

As anticipated, the selection of the electricity mix generally increases the climate change impact of each system, as illustrated in Figure S6. In instances where electricity constitutes a significant factor—such as AnMBR11, SBR1, SBR2, SBR3, and SBR4—the impacts associated with the underlying background choice are discernible, at times nearly doubling the total climate change impacts. Nonetheless, in scenarios where greenhouse gas (GHG) emissions are the primary contributing factor—namely AnMBR1 through AnMBR10—the variation in the electricity mix does not influence the results pertaining to contributions. Consequently, it can be concluded that although the choice of energy mix broadly affects the overall climate change impacts, the systems in which climate impact is predominantly driven by direct GHG emissions remain unaffected by the energy mix.

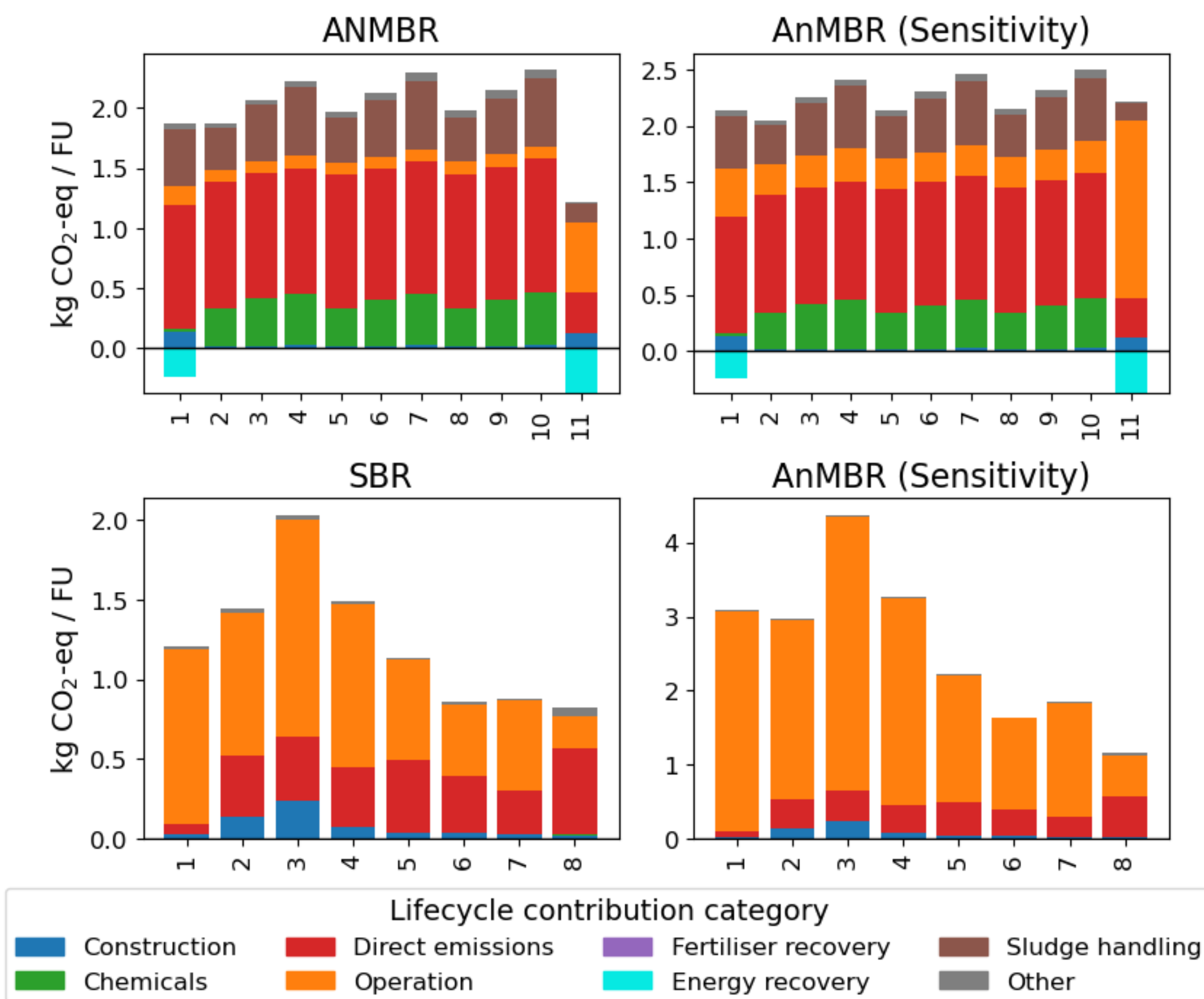

Figure S6. Contribution to climate change. Comparison of AnMBR and SBR using European electricity mix (left-hand side of the figure) and Chinese electricity mix (right-hand side of the figure)

Classified as Internal | Intern

References

Abbasi Moud, A. (2022). Polymer based flocculants: Review of water purification applications. *Journal of Water Process Engineering*, *48*, 102938. https://doi.org/10.1016/j.jwpe.2022.102938

Ahmed, Y., Yaakob, Z., Akhtar, P., & Sopian, K. (2015). Production of biogas and performance evaluation of existing treatment processes in palm oil mill effluent (POME). *Renewable and Sustainable Energy Reviews*, *42*, 1260–1278. https://doi.org/10.1016/j.rser.2014.10.073

Ashrafi, E., Mehrabani Zeinabad, A., Borghei, S. M., Torresi, E., & Muñoz Sierra, J. (2019). Optimising nutrient removal of a hybrid five-stage Bardenpho and moving bed biofilm reactor process using response surface methodology. *Journal of Environmental Chemical Engineering*, *7*(1), 102861. https://doi.org/10.1016/j.jece.2018.102861

Banayan Esfahani, E., Asadi Zeidabadi, F., Bazargan, A., & McKay, G. (2018). The Modified Bardenpho Process. In *Handbook of Environmental Materials Management* (pp. 1–43). Springer, Cham. https://doi.org/10.1007/978-3-319-58538-3_87-2

Bolto, B., & Gregory, J. (2007). Organic polyelectrolytes in water treatment. *Water Research*, *41*(11), 2301–2324. https://doi.org/10.1016/j.watres.2007.03.012

Çankaya, S., & Pekey, B. (2024). Evaluating the environmental and economic performance of biological and advanced biological wastewater treatment plants by life cycle

Classified as Internal | Intern

assessment and life cycle costing. *Environmental Monitoring and Assessment*, *196*(4), 373. https://doi.org/10.1007/s10661-024-12519-z

Chen, M., Xu, R., Wu, Y., Xiong, J., Keleş, S. Z., & Hankins, N. P. (2024). Application of polyelectrolytes for contaminant removal and recovery during water and wastewater treatment: A critical review. *Journal of Water Process Engineering*, *64*, 105528. https://doi.org/10.1016/j.jwpe.2024.105528

Cheng, Y., Tian, K., Xie, P., Ren, X., Li, Y., Kou, Y., Chon, K., Hwang, M.-H., & Ko, M.-H. (2022). Insights into the minimization of excess sludge production in micro-aerobic reactors coupled with a membrane bioreactor: Characteristics of extracellular polymeric substances. *Chemosphere*, *292*, 133434. https://doi.org/10.1016/j.chemosphere.2021.133434

Corbella, C., Puigagut, J., & Garfí, M. (2017). Life cycle assessment of constructed wetland systems for wastewater treatment coupled with microbial fuel cells. *Science of The Total Environment*, *584–585*, 355–362. https://doi.org/10.1016/j.scitotenv.2016.12.186

Corominas, L., Byrne, D. M., Guest, J. S., Hospido, A., Roux, P., Shaw, A., & Short, M. D. (2020). The application of life cycle assessment (LCA) to wastewater treatment: A best practice guide and critical review. *Water Research*, *184*, 116058. https://doi.org/10.1016/j.watres.2020.116058

Cui, L., Li, W., Zhang, Y., Wei, J., Lei, Y., Zhang, M., Pan, X., Zhao, X., Li, K., & Ma, W. (2016). Nitrogen Removal in a Horizontal Subsurface Flow Constructed Wetland Estimated

Classified as Internal | Intern

Using the First-Order Kinetic Model. *Water*, *8*(11), 514. https://doi.org/10.3390/w8110514

Czechowski, F., & Marcinkowski, T. (2006). Sewage sludge stabilisation with calcium hydroxide: Effect on physicochemical properties and molecular composition. *Water Research*, *40*(9), 1895–1905. https://doi.org/10.1016/j.watres.2006.02.023

Di Giacomo, G., & Romano, P. (2022). Evolution and Prospects in Managing Sewage Sludge Resulting from Municipal Wastewater Purification. *Energies*, *15*(15), 5633. https://doi.org/10.3390/en15155633

Doka, G. (2003). Life Cycle Inventories of Waste Treatment Services. *Swiss Centre for Life Cycle Inventories*, *ecoinvent report No. 13*.

Domini, M., Abbà, A., & Bertanza, G. (2022). Analysis of the variation of costs for sewage sludge transport, recovery and disposal in Northern Italy: A recent survey (2015–2021). *Water Science and Technology*, *85*(4), 1167–1175. https://doi.org/10.2166/wst.2022.040

Dutta, A., & Sarkar, S. (2015). Sequencing Batch Reactor for Wastewater Treatment: Recent Advances. *Current Pollution Reports*, *1*(3), 177–190. https://doi.org/10.1007/s40726-015-0016-y

Ebie, Y., Towprayoon, S., Chiemchaisri, C., Mander, Ü., Nogueira, S. F., Jamsranjav, B., Gordon, C., & Petrova, D. R. (2013). 2013 Supplement to the 2006 IPCC Guidelines for National Greenhouse Gas Inventories: Wetlands. *IPCC*, (Chaper 6).

Classified as Internal | Intern

European Comission. (2025). *European Commission | Fertiliser prices*. https://agridata.ec.europa.eu/extensions/DashboardFertiliser/FertiliserPrices.html

Eurostat. (2025, October). *Electricity price statistics*. https://ec.europa.eu/eurostat/statistics-explained/index.php?title=Electricity_price_statistics

Fallahnejad, M., Kranzl, L., Haas, R., Hummel, M., Müller, A., García, L. S., & Persson, U. (2024). District heating potential in the EU-27: Evaluating the impacts of heat demand reduction and market share growth. *Applied Energy*, *353*, 122154. https://doi.org/10.1016/j.apenergy.2023.122154

Foley, J., de Haas, D., Hartley, K., & Lant, P. (2010). Comprehensive life cycle inventories of alternative wastewater treatment systems. *Water Research*, *44*(5), 1654–1666. https://doi.org/10.1016/j.watres.2009.11.031

Guinée, J. B., Heijungs, R., & Huppes, G. (2004). Economic allocation: Examples and derived decision tree. *The International Journal of Life Cycle Assessment*, *9*(1), 23–33. https://doi.org/10.1007/BF02978533

Guo, C.-Z., Fu, W., Chen, X.-M., Peng, D.-C., & Jin, P.-K. (2013). Nitrogen-removal performance and community structure of nitrifying bacteria under different aeration modes in an oxidation ditch. *Water Research*, *47*(11), 3845–3853. https://doi.org/10.1016/j.watres.2013.04.005

Classified as Internal | Intern

Gupta, A. S., & Khatiwada, D. (2024). Investigating the sustainability of biogas recovery systems in wastewater treatment plants- A circular bioeconomy approach. *Renewable and Sustainable Energy Reviews*, *199*, 114447. https://doi.org/10.1016/j.rser.2024.114447

Hartley, K. J. (2008). Controlling sludge settleability in the oxidation ditch process. *Water Research*, *42*(6), 1459–1466. https://doi.org/10.1016/j.watres.2007.10.017

Högstrand, S., Wärff, C., Svanström, M., & Jönsson, K. (2024). Dynamic process simulation for life cycle inventory data acquisition – Environmental assessment of biological and chemical phosphorus removal. *Journal of Cleaner Production*, *479*, 144047. https://doi.org/10.1016/j.jclepro.2024.144047

Huang, X., Zhao, Y., Gao, B., Sun, S., Wang, Y., Li, Q., & Yue, Q. (2016). Polyacrylamide as coagulant aid with polytitanium sulfate in humic acid-kaolin water treatment: Effect of dosage and dose method. *Journal of the Taiwan Institute of Chemical Engineers*, *64*, 173–179. https://doi.org/10.1016/j.jtice.2016.04.011

Irvine, R. L., Ketchum Jr., L. H., & Asano, T. (1989). Sequencing batch reactors for biological wastewater treatment. *Critical Reviews in Environmental Control*, *18*(4), 255–294. https://doi.org/10.1080/10643388909388350

Jiménez-Benítez, A., Ruiz-Martínez, A., Ferrer, J., Ribes, J., Rogalla, F., & Robles, A. (2023). Life cycle costing of AnMBR technology for urban wastewater treatment: A case study based on a demo-scale AnMBR system. *Journal of Environmental Chemical Engineering*, *11*(3), 110267. https://doi.org/10.1016/j.jece.2023.110267

Jones, E. R., van Vliet, M. T. H., Qadir, M., & Bierkens, M. F. P. (2021). Country-level and gridded estimates of wastewater production, collection, treatment and reuse. *Earth System Science Data*, *13*(2), 237–254. https://doi.org/10.5194/essd-13-237-2021

Kanafin, Y. N., Kanafina, D., Malamis, S., Katsou, E., Inglezakis, V. J., Poulopoulos, S. G., & Arkhangelsky, E. (2021). Anaerobic Membrane Bioreactors for Municipal Wastewater Treatment: A Literature Review. *Membranes*, *11*(12), 967. https://doi.org/10.3390/membranes11120967

Kelessidis, A., & Stasinakis, A. S. (2012). Comparative study of the methods used for treatment and final disposal of sewage sludge in European countries. *Waste Management*, *32*(6), 1186–1195. https://doi.org/10.1016/j.wasman.2012.01.012

Khan, N. A., Singh, S., Ramamurthy, P. C., & Aljundi, I. H. (2024). Exploring nutrient removal mechanisms in column-type SBR with simultaneous nitrification and denitrification. *Journal of Environmental Management*, *349*, 119485. https://doi.org/10.1016/j.jenvman.2023.119485

Kitanou, S., Ayyoub, H., El-Ghzizel, S., Belhamidi, S., Taky, M., & Elmidaoui, A. (2021). Membrane bioreactor for domestic wastewater treatment: Energetic assessment. *Desalination and Water Treatment*, *240*, 55–62. https://doi.org/10.5004/dwt.2021.27675

Kong, Z., Wu, J., Rong, C., Wang, T., Li, L., Luo, Z., Ji, J., Hanaoka, T., Sakemi, S., Ito, M., Kobayashi, S., Kobayashi, M., Qin, Y., & Li, Y.-Y. (2021). Sludge yield and degradation of suspended solids by a large pilot-scale anaerobic membrane bioreactor for the

Classified as Internal | Intern

treatment of real municipal wastewater at 25 °C. *Science of The Total Environment*, *759*, 143526. https://doi.org/10.1016/j.scitotenv.2020.143526

Kulikowska, D., Klimiuk, E., & Drzewicki, A. (2007). BOD5 and COD removal and sludge production in SBR working with or without anoxic phase. *Bioresource Technology*, *98*(7), 1426–1432. https://doi.org/10.1016/j.biortech.2006.05.021

Li, P., Hu, X., Yuan, J., Sun, F., Li, P., Dong, W., Du, E., & Peng, M. (2024). Life cycle and environmental impact assessment of vegetation-activated sludge process (V-ASP) for decentralized wastewater treatment. *Current Research in Biotechnology*, *7*, 100172. https://doi.org/10.1016/j.crbiot.2023.100172

Li, Z., Deng, H., Yang, L., Zhang, G., Li, Y., & Ren, Y. (2018). Influence of potassium hydroxide activation on characteristics and environmental risk of heavy metals in chars derived from municipal sewage sludge. *Bioresource Technology*, *256*, 216–223. https://doi.org/10.1016/j.biortech.2018.02.013

Luo, Y., Yao, J., Wang, X., Zheng, M., Guo, D., & Chen, Y. (2020). Efficient municipal wastewater treatment by oxidation ditch process at low temperature: Bacterial community structure in activated sludge. *Science of The Total Environment*, *703*, 135031. https://doi.org/10.1016/j.scitotenv.2019.135031

Mainardis, M., Buttazzoni, M., & Goi, D. (2020). Up-Flow Anaerobic Sludge Blanket (UASB) Technology for Energy Recovery: A Review on State-of-the-Art and Recent Technological Advances. *Bioengineering*, *7*(2), 43. https://doi.org/10.3390/bioengineering7020043

Classified as Internal | Intern

Metcalf and Eddy, Tchobanoglous, G., Stensel, D. H., Tsuchihashi, R., Burton, F., Abu-Orf, M., Bowden, G., Pfrang, W., & Albert Einstein College of Medicine (Eds). (2014). *Wastewater engineering: Treatment and resource recovery* (Fifth Edition). McGraw-Hill Education.

Mohammed Nazeer Khan, Zuzana Dedova, & Arsou Arimi. (2021). *Techno-economic assessment of producing biodiesel from sewage* (INTERREG). https://vb.nweurope.eu/media/16740/1wpt1_deliverable_3_1_lipids_tea_final.pdf

Moral Pajares, E., Gallego Valero, L., & Román Sánchez, I. M. (2019). Cost of Urban Wastewater Treatment and Ecotaxes: Evidence from Municipalities in Southern Europe. *Water*, *11*(3), 423. https://doi.org/10.3390/w11030423

Morelli, B., Cashman, S., Ma, X. (Cissy), Garland, J., Turgeon, J., Fillmore, L., Bless, D., & Nye, M. (2018). Effect of Nutrient Removal and Resource Recovery on Life Cycle Cost and Environmental Impacts of a Small Scale Water Resource Recovery Facility. *Sustainability*, *10*(10), 3546. https://doi.org/10.3390/su10103546

Morera, S., Santana, M. V. E., Comas, J., Rigola, M., & Corominas, L. (2020). Evaluation of different practices to estimate construction inventories for life cycle assessment of small to medium wastewater treatment plants. *Journal of Cleaner Production*, *245*, 118768. https://doi.org/10.1016/j.jclepro.2019.118768

Osman, R. M., & Hodaifa, G. (2023). An overview of anaerobic membrane bioreactors: Current developments, fouling problems, and future prospects. *Journal of*

Classified as Internal | Intern

Environmental Chemical Engineering, *11*(6), 111482. https://doi.org/10.1016/j.jece.2023.111482

Pasciucco, F., Pecorini, I., & Iannelli, R. (2023). A comparative LCA of three WWTPs in a tourist area: Effects of seasonal loading rate variations. *Science of The Total Environment*, *863*, 160841. https://doi.org/10.1016/j.scitotenv.2022.160841

Pistocchi, A., Grizzetti, B., Nielsen, P. H., Parravicini, V., Steinmetz, H., Thornberg, D., & Vigiak, O. (2023). An Assessment of Options to Improve the Removal of Excess Nutrients from European Wastewater. *Water, Air, & Soil Pollution*, *234*(9), 595. https://doi.org/10.1007/s11270-023-06478-3

Pryce, D., Kapelan, Z., & Memon, F. A. (2022). Economic evaluation of a small wastewater treatment plant under different design and operation scenarios by life cycle costing. *Development Engineering*, *7*, 100103. https://doi.org/10.1016/j.deveng.2022.100103

Rattier, M., Jimenez, J. A., Miller, M. W., Dhanasekar, A., Willis, J., Keller, J., & Batstone, D. (2022). Long-term comparison of pilot UASB and AnMBR systems treating domestic sewage at ambient temperatures. *Journal of Environmental Chemical Engineering*, *10*(6), 108489. https://doi.org/10.1016/j.jece.2022.108489

Rezai, B., & Allahkarami, E. (2021). Chapter 2—Wastewater Treatment Processes—Techniques, Technologies, Challenges Faced, and Alternative Solutions. In R. R. Karri, G. Ravindran, & M. H. Dehghani (Eds), *Soft Computing Techniques in Solid*

Classified as Internal | Intern

Waste and Wastewater Management (pp. 35–53). Elsevier. https://doi.org/10.1016/B978-0-12-824463-0.00004-5

Scrinzi, D., Ferrentino, R., Baù, E., Fiori, L., & Andreottola, G. (2023). Sewage Sludge Management at District Level: Reduction and Nutrients Recovery via Hydrothermal Carbonization. *Waste and Biomass Valorization*, *14*(8), 2505–2517. https://doi.org/10.1007/s12649-022-01943-2

Seiple, T. E., Coleman, A. M., & Skaggs, R. L. (2017). Municipal wastewater sludge as a sustainable bioresource in the United States. *Journal of Environmental Management*, *197*, 673–680. https://doi.org/10.1016/j.jenvman.2017.04.032

S.R. Nikou, N., Mc Nabola, A., & Gill, L. (2025). Techno-economic and environmental analysis of Modified Ludzack-Ettinger (MLE) process for enhanced energy efficiency and recovery. *Journal of Water Process Engineering*, *74*, 107728. https://doi.org/10.1016/j.jwpe.2025.107728

Srivastava, N., & Chattopadhyay, J. (2021). 25—Bacterial community structure, composition and their role in biological wastewater treatment reactors plants. In M. P. Shah & S. Rodriguez-Couto (Eds), *Wastewater Treatment Reactors* (pp. 583–597). Elsevier. https://doi.org/10.1016/B978-0-12-823991-9.00020-4

Stanbury, P. F., Whitaker, A., & Hall, S. J. (2017). Chapter 11—Effluent treatment. In P. F. Stanbury, A. Whitaker, & S. J. Hall (Eds), *Principles of Fermentation Technology (Third Edition)* (pp. 687–723). Butterworth-Heinemann. https://doi.org/10.1016/B978-0-08-099953-1.00011-9

Classified as Internal | Intern

Tantak, N., Chandan, N., & Raina, P. (2014). Chapter 9 - An Introduction to Biological Treatment and Successful Application of the Aqua EMBR System in Treating Effluent Generated from a Chemical Manufacturing Unit: A Case Study. In V. V. Ranade & V. M. Bhandari (Eds), *Industrial Wastewater Treatment, Recycling and Reuse* (pp. 369–397). Butterworth-Heinemann. https://doi.org/10.1016/B978-0-08-099968-5.00009-X

Tarpani, R. R. Z., Alfonsín, C., Hospido, A., & Azapagic, A. (2020). Life cycle environmental impacts of sewage sludge treatment methods for resource recovery considering ecotoxicity of heavy metals and pharmaceutical and personal care products. *Journal of Environmental Management*, *260*, 109643. https://doi.org/10.1016/j.jenvman.2019.109643

Wang, C., Ng, T. C. A., & Ng, H. Y. (2021). Comparison between novel vibrating ceramic MBR and conventional air-sparging MBR for domestic wastewater treatment: Performance, fouling control and energy consumption. *Water Research*, *203*, 117521. https://doi.org/10.1016/j.watres.2021.117521

Wang, Y., & Serventi, L. (2019). Sustainability of dairy and soy processing: A review on wastewater recycling. *Journal of Cleaner Production*, *237*, 117821. https://doi.org/10.1016/j.jclepro.2019.117821

Xie, W.-M., Zhang, R., Li, W.-W., Ni, B.-J., Fang, F., Sheng, G.-P., Yu, H.-Q., Song, J., Le, D.-Z., Bi, X.-J., Liu, C.-Q., & Yang, M. (2011). Simulation and optimization of a full-scale

Classified as Internal | Intern

Carrousel oxidation ditch plant for municipal wastewater treatment. *Biochemical Engineering Journal*, *56*(1), 9–16. https://doi.org/10.1016/j.bej.2011.04.010

Yan, Y., & Xu, J. (2014). Improving Winter Performance of Constructed Wetlands for Wastewater Treatment in Northern China: A Review. *Wetlands*, *34*(2), 243–253. https://doi.org/10.1007/s13157-013-0444-7

Yoshida, H., Mønster, J., & Scheutz, C. (2014). Plant-integrated measurement of greenhouse gas emissions from a municipal wastewater treatment plant. *Water Research*, *61*, 108–118. https://doi.org/10.1016/j.watres.2014.05.014

Zhang, Y., Zhang, W., Wang, H., Wu, Y., & Liu, B. (2023). N, P and C removal simultaneously and microbial population numbers in a cyclic activated sludge system treating village and township domestic wastewater by altering the cycle times. *Water Science and Technology*, *88*(9), 2271–2283. https://doi.org/10.2166/wst.2023.342

Classified as Internal | Intern
